# Comparison of three longitudinal analysis models for the health-related quality of life in oncology: a simulation study

**DOI:** 10.1186/s12955-014-0192-2

**Published:** 2014-12-31

**Authors:** Amélie Anota, Antoine Barbieri, Marion Savina, Alhousseiny Pam, Sophie Gourgou-Bourgade, Franck Bonnetain, Caroline Bascoul-Mollevi

**Affiliations:** Quality of Life in Oncology National Platform, Besançon, France; Methodological and Quality of Life in Oncology Unit, EA 3181, University Hospital of Besançon, Besançon, France; Biostatistic unit, Institut régional du Cancer de Montpellier (ICM) - Val d’Aurelle, Montpellier, France; Institut de Mathématiques et de Modélisation de Montpellier, University of Montpellier 2, Montpellier, France; INSERM, Clinical and EpidemiologicalResearch Unit (CIC-EC 7) – CTD INCa, Institut Bergonié, Bordeaux, France; INSERM CIC-EC7 Axe Cancer, Université de Bordeaux, Bordeaux, France

**Keywords:** Longitudinal analysis, Statistical methods, Health-related quality of life, Oncology clinical trials

## Abstract

**Background:**

Health-Related Quality of Life (HRQoL) is an important endpoint in oncology clinical trials aiming to investigate the clinical benefit of new therapeutic strategies for the patient. However, the longitudinal analysis of HRQoL remains complex and unstandardized. There is clearly a need to propose accessible statistical methods and meaningful results for clinicians. The objective of this study was to compare three strategies for longitudinal analyses of HRQoL data in oncology clinical trials through a simulation study.

**Methods:**

The methods proposed were: the score and mixed model (SM); a survival analysis approach based on the time to HRQoL score deterioration (TTD); and the longitudinal partial credit model (LPCM). Simulations compared the methods in terms of type I error and statistical power of the test of an interaction effect between treatment arm and time. Several simulation scenarios were explored based on the EORTC HRQoL questionnaires and varying the number of patients (100, 200 or 300), items (1, 2 or 4) and response categories per item (4 or 7). Five or 10 measurement times were considered, with correlations ranging from low to high between each measure. The impact of informative missing data on these methods was also studied to reflect the reality of most clinical trials.

**Results:**

With complete data, the type I error rate was close to the expected value (5%) for all methods, while the SM method was the most powerful method, followed by LPCM. The power of TTD is low for single-item dimensions, because only four possible values exist for the score. When the number of items increases, the power of the SM approach remained stable, those of the TTD method increases while the power of LPCM remained stable. With 10 measurement times, the LPCM was less efficient. With informative missing data, the statistical power of SM and TTD tended to decrease, while that of LPCM tended to increase.

**Conclusions:**

To conclude, the SM model was the most powerful model, irrespective of the scenario considered, and the presence or not of missing data. The TTD method should be avoided for single-item dimensions of the EORTC questionnaire. While the LPCM model was more adapted to this kind of data, it was less efficient than the SM model. These results warrant validation through comparisons on real data.

**Electronic supplementary material:**

The online version of this article (doi:10.1186/s12955-014-0192-2) contains supplementary material, which is available to authorized users.

## Background

Health-Related Quality of Life (HRQoL) is an important endpoint in oncology clinical trials aiming to investigate the clinical benefit of new therapeutic strategies for the patient and health care system [1]. However, the longitudinal analysis of HRQoL remains complex and unstandardized. To date, no recommendations have been made on how to analyze longitudinal HRQoL data in oncology, which is a key issue to facilitate comparison of results between trials. Moreover, there is a clear need to propose accessible statistical methods and meaningful results for clinicians.

HRQoL is a subjective endpoint that is not directly observable, and it is therefore considered as a latent trait. Patients’ HRQoL level is generally estimated by administering validated questionnaires given to the patients at different time points for a longitudinal approach.

In oncology clinical trials, one of the most widely used questionnaires is the European Organization for Research and Treatment of Cancer (EORTC) Quality of Life Questionnaire (QLQ-C30), which is a validated, self-administered questionnaire specific to cancer [2]. The QLQ-C30 is a multidimensional questionnaire that makes it possible to evaluate several HRQoL domains (functional and symptomatic) specific to cancer. Each dimension is evaluated through one or more polytomous items. A score is estimated for each dimension according to specific scoring guidelines [3]. HRQoL questionnaires are administered to the patients several times, depending on the therapeutic setting: generally, at baseline (before randomization), during treatment (e.g. at each chemotherapy cycle), at the end of the study and/or repeatedly during the follow-up until tumor progression or death. The objective is to analyze the course of the patient HRQoL over time. Given this longitudinal assessment, data are often missing, particularly in the advanced or metastatic settings [4].

Three types of missing data exist according to Little and Rubin’s classification [5]. If the missing data are not dependent on either past or present observed variables (such as HRQoL level), then they are considered as missing completely at random (MCAR). For example, a patient can forget to complete an item or a questionnaire at one measurement time. Missing data are missing at random (MAR) if they are not dependent on the present HRQoL level, but can be explained by a previously observed variable (previously observed HRQoL level or other clinical or socio-demographic characteristic of the patient). For example, the age of a patient may explain their reluctance to answer a particular question. Finally, missing data are missing not at random (MNAR) if they are dependent on the present, unobserved HRQoL level. For example, if the patient did not complete a questionnaire due to his/her altered health status, it can reflect a deterioration of his/her HRQoL level. MCAR and MAR missing data are non-informative and thus may not induce a bias in the analysis. In contrast, the MNAR profile corresponds to informative data and can bias the results if it is not adequately taken into account in the longitudinal analysis method. In oncology clinical trials and especially in advanced cancers, missing data are most often MNAR [6].

Missing data can be intermittent or monotone. Intermittent missing items correspond to patients who fail to complete one or more items in a given questionnaire [7]. Entire forms may also be missing if the patient cannot fill out the HRQoL questionnaire at a given measurement time (intermittent missing form) [8]. In both these cases, the patient will complete another questionnaire and remains present in the study, since other HRQoL data are available for that patient after the occurrence of this intermittent missing data. Conversely, when a patient drops out of the study prematurely, generally due to a deterioration of health state or death, this corresponds to monotone missing data [9]. In this case, no further data are available after the patient drops out. The risk of this situation is that only patients with the highest HRQoL level be will analyzed. The impact of missing data in longitudinal analysis has often been explored in previous studies [10].

The longitudinal analysis of HRQoL data is generally performed according to the Classical Test Theory (CTT). In the CTT, the score constructed from the item answers is considered as a good representation of the “true” HRQoL level. Therefore, longitudinal analysis is based on this score, considering that it is a semi-quantitative measure, even if only one item is used to construct the score. Item Response Theory (IRT) is another approach, in which items play a key role [11]. IRT models link the item responses to the latent trait by a probabilistic model, generally with a logistic link. An important class of IRT models is the Rasch-family models [12].

Some previous simulation studies have compared CTT and IRT approaches for the longitudinal analyses of patient-reported outcomes such as HRQoL [13-16]. These studies highlighted the similar performance of both approaches in the context of complete data [13] and in the presence of monotone missing data [14]. In the presence of informative intermittent missing data, the Rasch-family models seem to be more efficient than CTT and, in particular, provide high statistical power [15]. However, all these studies were performed on dichotomous items and restricted to three measurement times. Dichotomous items are rarely used in HRQoL questionnaires. The EORTC HRQoL questionnaires, like most other HRQoL questionnaires, are built on a Likert scale with polytomous items. Moreover, in oncology clinical trials, more than three measurement times are generally planned. Therefore, there exists a pressing need to compare these two approaches in the context of polytomous items with more than three measurement times. These previous simulation studies also focused on the effects of time or treatment arm [13-16]. In randomized clinical trials, HRQoL level is supposed to be equal in both treatment arms at baseline. To detect a different effect, we investigate whether there is a significant difference between arms in HRQoL over time, using an interaction parameter between treatment arm and time. While the interaction between time and treatment has often been explored on real data in oncology clinical trials [17], it has never been investigated in longitudinal HRQoL simulations, to the best of our knowledge.

In previous studies, the CTT-based approach evaluated was the score and mixed model (SM). This method is the most widely used for longitudinal analyses. However, in oncology clinical trials, a time to event approach, i.e. the so-called time to HRQoL score deterioration (TTD) has come to be used extensively [18-21]. This method has the advantage of producing meaningful results for clinicians as compared to IRT models, and more generally, mixed models. No study to date has compared TTD to SM and IRT models.

In this context, the objective of this study was to compare, through a simulation study, three statistical methods for analyzing longitudinal HRQoL data in oncology clinical trials, namely:

- two CTT-based approaches, namely the SM model and the TTD approach;
- and a longitudinal IRT model for polytomous items called the Longitudinal Partial Credit Model (LPCM).

Simulations compared the methods in terms of type I error rate and statistical power of the test for an interaction effect between treatment arm and time. To reflect the reality of most clinical trials, the impact of informative missing data on these methods was also studied, with the implementation of both intermittent and monotone missing data, depending on the patients’ HRQoL level (MNAR profile).

## Methods

### Longitudinal analysis models for health-related quality of life

#### Score and mixed model

In CTT, the observed score is considered to be closed to the real HRQoL level, i.e. the relationship between the observed score and the “true” score is linear.

The SM model, based on the CTT approach, involves applying a linear mixed model to the observed HRQoL scores computed at each measurement time.

We considered a model with two fixed effects: an interaction effect between the treatment arm and time (difference in HRQoL changes between both treatments); and a time effect (course of HRQoL over time). Moreover, we added a random effect on patient (individual deviance from average intercept) and time (individual deviance from average time effect) with an unstructured covariance matrix. The formula of the model considered is given in Additional file 1. Random effects models give unbiased results in case of MAR. For MNAR, pattern mixture models can be used [22].

Parameters were estimated using the Maximum Likelihood method, which is based on the Newton–Raphson algorithm. The model was implemented using SAS software version 9.3 (SAS Institute Inc., Cary, NC, USA) with PROC MIXED.

#### Time to health-related quality of life score deterioration

The TTD approach is also based on the observed score and relies on the definition of the minimal clinically important difference (MCID) in order to be effective from a clinical point of view. Several definitions of TTD have been proposed according to the therapeutic situation and cancer site. Events can be defined according to the chosen reference score, MCID, missing scores, including all-cause death or not. Given the multiplicity of possible definitions of TTD, a standardization of the longitudinal analysis of HRQoL data in oncology according to the TTD approach has been proposed [23]. Accordingly, four main definitions have been retained in the present paper, in conformity with these recommendations.

The most intuitive definition of the TTD is the time from inclusion-randomization in the study to a first deterioration of at least one MCID unit as compared to baseline score [24]. Patients with no deterioration before their drop-out are censored at the time of the last HRQoL assessment.

The observed deterioration can be definitive or not. In the palliative setting, it is more relevant to study the time until definitive HRQoL score deterioration (TUDD). TUDD reflects the deterioration of the patient’s health status (which is stable over time) and represents an absorbing state. TUDD has been defined as the time from inclusion-randomization in the study to a first deterioration of at least one MCID unit as compared to the baseline score, with no further improvement of more than one MCID unit as compared to baseline, or if the patient drops out after deterioration, resulting in missing data [18].

In the published definitions, the reference score is the baseline score. However, other scores can be chosen as a reference, such as the best previous score. Indeed, the baseline score is not necessarily the reference score for the patient in the case of a change in the patient’s internal standard, illustrating one component of a response shift effect [23,25,26]. Therefore, both options were retained in order to study their impact on this approach.

Regarding the EORTC HRQoL questionnaires, a 5-point deterioration in HRQoL scores is generally considered as the MCID [27]. The MCID was thus fixed at 5 points.

Table 1 summarizes the four definitions of TTD/TUDD retained in our study.

**Table 1:** **Summary of the definitions of time to quality of life score deterioration approach retained for the simulation study**

MCID 5-point	Reference score	Deterioration
TTD ≥ 5-points	Baseline	Not definitive
TUDD ≥ 5-points with no further improvement 5 points as compared to reference score	Baseline	Definitive
TTD ≥ 5-points	Best previous score	Not definitive
TUDD ≥ 5-points with no further improvement 5 points as compared to reference score and	Best previous score	Definitive

MCID: Minimal Clinically Important Difference.

TTD: Time To Deterioration.

TUDD: Time Until Definitive Deterioration.

Furthermore, a high score corresponds to a high level of functioning on a functional scale, but corresponds to strong presence of symptoms for a symptomatic scale. Therefore, “deterioration” was defined as a decrease on the functional scale or global health status dimension, and as an increase on the symptomatic scale.

In the basic TTD/TUDD approach, intermittent missing data were ignored, and we considered that the patient’s HRQoL level remained unchanged since the last available HRQoL assessment.

The TTD and TUDD estimations were calculated using the Kaplan-Meier method [28].

These definitions of TTD and TUDD were implemented using SAS software.

#### Longitudinal mixed partial credit model

An important family of IRT models is the Rasch-family models. Despite the interesting properties of these models, such as specific objectivity, they are still rarely applied for the longitudinal analysis of HRQoL data. To date, few investigations are ongoing using this technique in clinical oncology [29,30].

The Partial Credit Model (PCM) is a Rasch-family model adapted to polytomous items [31]. The PCM models the probability that one individual *n* will choose the response category *k* among the *m*_*j*_ possible responses for the item *j* (i.e. generalized linear mixed model with a multinomial logit link function) given the latent trait *θ*_*n*_ and the category difficulty parameters ${\delta}_{j,1},\dots, {\delta}_{j,{m}_j}$ for the item *j* (see Additional file 1 for the formula).

As with all Rasch-family models, the PCM relies on three fundamental assumptions, namely unidimensionality of the latent trait, monotonicity and local independence of the items conditionally to the latent trait.

In this study, a longitudinal extension of the PCM to mixed-effect regression models was used and called the Longitudinal PCM (LPCM).

Regarding this model, we considered a model with two fixed effects; namely an interaction between treatment and time; and a time effect. Moreover, we added a random effect on patient and time with an unstructured covariance matrix (see Additional file 1 for the formula).

This model was implemented using SAS software, using PROC NLMIXED.

### Simulation algorithm

#### Complete data

The complete datasets were simulated in two steps.

The first step corresponded to the simulation of the latent trait *θ*_*n*_ (*θ*_1_, *θ*_2_, *θ*_3_, *θ*_4_, *θ*_5_)*′* for 5 measurement times, for example, and for *n =* 1,…, *N* patients. This simulation was performed for each treatment arm (0/1) with *N*/2 patients per arm. The latent trait followed a multivariate normal distribution *N*_5_ (*μ*^0^, Σ) with mean *μ*^0^ = (*μ*_1_, *μ*_2_, *μ*_3_, *μ*_4_, *μ*_5_)*′* for the control arm (0) and first-order autoregressive covariance matrix $$ {\displaystyle \sum ={\sigma}^2}\left(\begin{array}{ccccc}\hfill 1\hfill & \hfill \rho \hfill & \hfill {\rho}^2\hfill & \hfill {\rho}^3\hfill & \hfill {\rho}^4\hfill \\ {}\hfill \rho \hfill & \hfill 1\hfill & \hfill \rho \hfill & \hfill {\rho}^2\hfill & \hfill {\rho}^3\hfill \\ {}\hfill {\rho}^2\hfill & \hfill \rho \hfill & \hfill 1\hfill & \hfill \rho \hfill & \hfill {\rho}^2\hfill \\ {}\hfill {\rho}^3\hfill & \hfill {\rho}^2\hfill & \hfill \rho \hfill & \hfill 1\hfill & \hfill \rho \hfill \\ {}\hfill {\rho}^4\hfill & \hfill {\rho}^3\hfill & \hfill {\rho}^2\hfill & \hfill \rho \hfill & \hfill 1\hfill \end{array}\right) $$. In the first-order autoregressive matrix, the correlation between HRQoL measures was assumed to decrease over time [13-15]. We fixed *σ*^2^ = 1. For the experimental arm (1), the latent trait was assumed to follow a multivariate normal distribution *N*_5_ (*μ*^1^, Σ) with mean *μ*^1^ = *μ*^0^ + Δ and with the same covariance matrix. Δ represented the treatment arm effect. In case of no treatment arm effect, Δ =0, otherwise Δ ≠ 0.

The second step of the complete dataset simulation corresponded to the determination of the item answers. The patients’ responses to the items were obtained with a LPCM in order to respect the three assumptions of the Rasch-family models [32]. Category difficulty parameters were fixed to estimated standard normal-distribution quantiles and were similar for all items.

Several simulations scenarios were explored based on the EORTC HRQoL questionnaires [2] and with variations in the number of patients (100, 200 or 300), items (1, 2 or 4) and response categories per item (4 or 7).

The value of the category difficulty parameters were as follows:

- *δ*_1_ = −0.7; *δ*_2_ = 0 and *δ*_3_ = 0.7 for items with 4 response categories,
- *δ*_1_ = −1; *δ*_2_ = −0.6; *δ*_3_ = −0.2; *δ*_4_ = 0.2; *δ*_5_ = 0.6 and *δ*_6_ = 1 for items with 7 response categories.

The simulations were performed with 4 or 7 response categories per item in order to reflect the construction of the EORTC HRQoL questionnaires [2]. Simulations with 7 response categories per item were only performed with 2 items to illustrate the Global Health Status dimension of the QLQ-C30 questionnaire and only with 200 patients.

At each measurement time, a score was then computed for each patient according to the recommendations of the EORTC HRQoL questionnaires for a symptomatic scale or Global Health Status scale [3]. The score *Y*_*n*_ of the *n-th* patient for a dimension composed of *I* items is then equal to $\left(\left(\frac{1}{I}{\displaystyle {\sum}_i{X}_i}\right)-1\right)\times \frac{100}{r}$, with r as the difference between the highest and the lowest possible response to the items.

Five or 10 measurement times were considered with a weak (0.4), moderate (0.7) or strong (0.9) correlation between each measure. Each scenario was simulated with a time effect equal to:

- *μ*^0^ = (-0.4 -0.2 0 0.2 0.4) for 5 measurement times, and
- *μ*^0^ = (-0.4 -0.3 -0.2 -0.1 0 0.1 0.2 0.3 0.4 0.5) for 10 measurement times.

As the mean of the latent trait increased over time, we considered that the score observed corresponded to a symptomatic scale. In this way, in the TTD approach, the deterioration was observed when the score increased.

Each scenario was performed with a treatment arm effect (Δ ≠ 0) or not (Δ =0). Different treatment arm effects were tested and we retained the following effects:

- Δ_1_ = 0 for the first measurement time *t* = 1,
- Δ_*t*_ = 0.4, ∀ *t* > 1.

#### Generation of missing data

Simulations were then repeated with missing data generated from the complete datasets.

Only simulation of an MNAR profile was performed, i.e. whereby patients with lower HRQoL levels were more likely to present missing data [33]. The complete algorithm for generating missing data is presented in Additional file 2. In order to reflect the reality of most clinical trials, both intermittent and monotone missing data were simulated.

For datasets with 5 measurement times:

- intermittent missing data were simulated on the second and third times
- and monotone missing data on the fourth and fifth times.

For datasets with 10 measurement times:

- intermittent missing data were simulated from the second to the sixth measure
- and monotone missing data from the seventh to the tenth measure.

In both cases, no missing data were generated at baseline.

Two types of intermittent missing data were considered: intermittent missing forms and intermittent missing items. Regarding intermittent missing forms, simulation of missing data was performed at each measurement time: if patient *i* presents missing data at time *t*, then all items of the dimension are missing for that patient at time *t*. For CTT-based methods (SM and TTD), simple imputation of missing items was performed by using the mean of the answered items, provided at least half of the items were answered by the patient, in accordance with the recommendation of the EORTC HRQoL questionnaires (personal mean score) to estimate the score.

Analyses were first conducted with both intermittent missing forms and drop-out, and then with intermittent missing items and drop-out. Analyses were conducted with a proportion *π*^(*t*)^ of missing data at each measurement time *t* equal to 10%, 20% or 30%.

#### Criteria for comparing the statistical methods

The type I error rate was estimated under the null hypothesis *H*_0_ of the absence of a treatment arm effect (Δ =0). It was calculated as the proportion of rejection of *H*_0_ under the null hypothesis.

The statistical power of the test of an interaction effect between treatment arm and time was estimated under the alternative hypothesis *H*_1_ of the presence of a treatment arm effect (Δ_1_ = 0; Δ_*t*_ = 0.4, ∀ *t* > 1). It was calculated as the proportion of rejection of *H*_0_ under the alternative hypothesis *H*_1_. The Wald and log-rank tests were used respectively for mixed models and survival analyses based on the TTD to test the rejection of the null hypothesis. Each scenario was simulated 500 times in order to have accurate estimations of the type I error rate and statistical power.

In order to clarify all the scenarios investigated, the parameters and their corresponding values are summarized in Table 2.

**Table 2:** **Summary of the parameters used in the simulation study and the corresponding values**

**Parameters**	**Values**
Number of time points	5 or 10
Time effect	Linear: *μ*_0_ = −0.4 and *μ*_*end*_ = −0.4
Arm effect	Δ = 0 for T ≥ T_0_ or Δ = 0 at T_0_; Δ = 0.4 for T > T_0_
Correlation between HRQoL measures	0.4; 0.7; 0.9
Number of Patients	100; 200; 300
Items	1; 2; 4
Response Categories per item	4; 7 (only for 2 items, 200 patients)
Percentage of missing data at each follow-up	0; 10%; 20%; 30%

## Results

### Complete data

With complete data, the type I error rate was close to the expected value (5%) for all methods (Table 3). The SM method was the most powerful method, irrespective of the parameter values in each scenario (Table 4). The statistical power of the TTD/TUDD approach was low, especially for single-item dimensions. The statistical power of the LPCM was intermediate, falling between that of the SM and that of the TTD/TUDD approach. For example, with *N* = 300 patients, *I* = 1 item, *ρ* = 0.4 and 5 measures, the power of the SM method, TTD vs baseline (“TTD baseline”) and LPCM was around 93%, 22% and 92% respectively. When the number of items increased, the statistical power of the SM approach remained stable, those of TTD/TUDD approach increased while the power of the LPCM remained stable. For 10 measurement times, the LPCM method was less powerful than for 5 measurement times. For example, when *N* = 300 patients, *I* = 4 items, ρ = 0.7 and with 5 measurement times, the power of the LPCM method was around 79%, while that of the SM method was around 96%. With 10 measurement times and the same value for all other parameters, the power of the LPCM method decreased to 52% while that of the SM method was around 99%. The power of the SM method and the TTD/TUDD approaches increased for items with 7 response categories as compared to those with 4 response categories, while the power of LPCM decreased slightly. When the correlation between measures increased, the power of the SM method tended to decrease overall, while that of the TTD/TUDD approach tended to increase (although the power values remained low) and the power of LPCM tended to decrease.

**Table 3:** **Type I error rate of the test of interaction between treatment arm and time, for simulations with complete data**

**N**	**I**	**J**	**ρ**	**5 measures**						**10 measures**					
				**SM**	**TTD baseline**	**TTD best**	**TUDD baseline**	**TUDD best**	**LPCM**	**SM**	**TTD baseline**	**TTD best**	**TUDD baseline**	**TUDD best**	**LPCM**
100	1	4	0.4	0.056	0.076	0.062	0.062	0.068	0.068	0.042	0.058	0.046	0.070	0.060	0.100
			0.7	0.048	0.028	0.058	0.052	0.060	0.048	0.032	0.050	0.066	0.042	0.040	0.044
			0.9	0.062	0.032	0.040	0.056	0.048	0.064	0.044	0.066	0.054	0.056	0.076	0.046
	2	4	0.4	0.062	0.058	0.048	0.056	0.064	0.056	0.054	0.046	0.042	0.046	0.060	0.050
			0.7	0.048	0.064	0.064	0.056	0.062	0.048	0.048	0.058	0.048	0.052	0.064	0.044
			0.9	0.054	0.054	0.054	0.050	0.044	0.066	0.040	0.066	0.058	0.056	0.056	0.036
	4	4	0.4	0.080	0.062	0.070	0.064	0.072	0.070	0.052	0.048	0.060	0.070	0.064	0.046
			0.7	0.078	0.040	0.048	0.052	0.068	0.072	0.048	0.060	0.060	0.050	0.064	0.052
			0.9	0.078	0.046	0.046	0.056	0.060	0.070	0.042	0.048	0.056	0.068	0.048	0.046
200	1	4	0.4	0.038	0.050	0.046	0.046	0.040	0.054	0.068	0.052	0.048	0.072	0.050	0.138
			0.7	0.056	0.064	0.042	0.054	0.062	0.054	0.054	0.052	0.064	0.054	0.054	0.058
			0.9	0.040	0.034	0.052	0.058	0.048	0.034	0.034	0.038	0.036	0.042	0.042	0.046
	2	4	0.4	0.046	0.060	0.048	0.046	0.042	0.046	0.044	0.064	0.064	0.068	0.054	0.048
			0.7	0.054	0.072	0.072	0.074	0.060	0.054	0.032	0.054	0.050	0.048	0.044	0.046
			0.9	0.068	0.054	0.060	0.046	0.046	0.066	0.040	0.044	0.058	0.058	0.044	0.042
		7	0.4	0.040	0.036	0.046	0.034	0.046	0.040	0.050	0.056	0.040	0.062	0.054	0.054
			0.7	0.044	0.046	0.050	0.056	0.056	0.040	0.038	0.066	0.058	0.056	0.054	0.042
			0.9	0.062	0.046	0.052	0.054	0.042	0.056	0.042	0.058	0.040	0.060	0.054	0.040
	4	4	0.4	0.042	0.056	0.050	0.052	0.060	0.036	0.048	0.032	0.056	0.052	0.046	0.042
			0.7	0.044	0.050	0.060	0.052	0.052	0.052	0.038	0.052	0.058	0.058	0.066	0.056
			0.9	0.054	0.042	0.050	0.052	0.042	0.050	0.030	0.054	0.048	0.064	0.054	0.042
300	1	4	0.4	0.046	0.054	0.064	0.052	0.072	0.058	0.050	0.052	0.046	0.050	0.058	0.108
			0.7	0.076	0.058	0.066	0.068	0.066	0.074	0.034	0.042	0.044	0.054	0.054	0.046
			0.9	0.038	0.050	0.040	0.050	0.052	0.034	0.034	0.058	0.072	0.074	0.076	0.036
	2	4	0.4	0.046	0.072	0.058	0.082	0.058	0.054	0.034	0.056	0.054	0.072	0.054	0.062
			0.7	0.044	0.058	0.046	0.044	0.052	0.046	0.040	0.054	0.050	0.052	0.058	0.038
			0.9	0.052	0.060	0.064	0.050	0.046	0.054	0.050	0.064	0.062	0.038	0.050	0.044
	4	4	0.4	0.040	0.062	0.068	0.042	0.044	0.064	0.038	0.042	0.060	0.048	0.062	0.040
			0.7	0.050	0.054	0.062	0.054	0.050	0.060	0.044	0.050	0.060	0.048	0.056	0.048
			0.9	0.044	0.066	0.062	0.044	0.052	0.038	0.036	0.044	0.048	0.028	0.044	0.048

The methods compared are the Score and Mixed Model (SM), Longitudinal Partial Credit Model (LPCM), Time to HRQoL score deterioration as compared to the baseline score (TTD baseline) or the best previous score (TTD best) and time until definitive deterioration of the HRQoL score as compared to the baseline score (TUDD baseline) or the best previous score (TUDD best) for different values of sample size (N), items (I), response category per item (J) and correlations between HRQoL measures (ρ).

**Table 4:** **Power of the test of interaction between treatment arm and time, for simulations with complete data**

**N**	**I**	**J**	**ρ**	**5 measurement times**						**10 measurement times**					
				**SM**	**TTD baseline**	**TTD best**	**TUDD baseline**	**TUDD best**	**LPCM**	**SM**	**TTD baseline**	**TTD best**	**TUDD baseline**	**TUDD best**	**LPCM**
100	1	4	0.4	0.518	0.118	0.102	0.130	0.100	0.472	0.784	0.134	0.132	0.120	0.058	0.654
			0.7	0.418	0.148	0.104	0.156	0.126	0.388	0.578	0.114	0.110	0.118	0.082	0.484
			0.9	0.404	0.148	0.124	0.174	0.142	0.380	0.414	0.124	0.126	0.130	0.084	0.352
	2	4	0.4	0.590	0.182	0.146	0.218	0.138	0.528	0.874	0.172	0.158	0.192	0.080	0.726
			0.7	0.488	0.214	0.162	0.208	0.162	0.432	0.628	0.188	0.180	0.192	0.096	0.354
			0.9	0.414	0.226	0.150	0.216	0.134	0.394	0.466	0.206	0.164	0.210	0.096	0.290
	4	4	0.4	0.680	0.260	0.168	0.204	0.128	0.412	0.916	0.236	0.190	0.246	0.090	0.396
			0.7	0.550	0.290	0.214	0.246	0.148	0.394	0.664	0.282	0.228	0.258	0.116	0.214
			0.9	0.496	0.394	0.290	0.364	0.210	0.428	0.428	0.400	0.284	0.314	0.150	0.228
200	1	4	0.4	0.812	0.212	0.162	0.240	0.142	0.778	0.970	0.140	0.168	0.182	0.060	0.858
			0.7	0.644	0.208	0.168	0.204	0.148	0.622	0.872	0.170	0.184	0.216	0.066	0.798
			0.9	0.644	0.234	0.176	0.256	0.160	0.612	0.670	0.194	0.152	0.204	0.082	0.572
	2	4	0.4	0.894	0.296	0.232	0.304	0.148	0.830	0.992	0.272	0.246	0.328	0.116	0.952
			0.7	0.760	0.350	0.262	0.344	0.214	0.678	0.934	0.326	0.242	0.334	0.114	0.618
			0.9	0.720	0.416	0.324	0.440	0.276	0.710	0.726	0.398	0.316	0.400	0.156	0.494
		7	0.4	0.936	0.346	0.270	0.328	0.214	0.832	0.999	0.362	0.324	0.366	0.096	0.900
			0.7	0.826	0.468	0.392	0.458	0.284	0.678	0.926	0.444	0.358	0.360	0.124	0.458
			0.9	0.810	0.580	0.442	0.614	0.400	0.788	0.726	0.600	0.506	0.536	0.218	0.458
	4	4	0.4	0.954	0.402	0.292	0.364	0.156	0.722	0.996	0.370	0.288	0.366	0.118	0.672
			0.7	0.812	0.552	0.394	0.456	0.236	0.606	0.934	0.484	0.362	0.380	0.142	0.368
			0.9	0.796	0.678	0.518	0.632	0.388	0.760	0.728	0.608	0.510	0.494	0.196	0.374
300	1	4	0.4	0.928	0.218	0.180	0.274	0.160	0.916	0.998	0.202	0.236	0.234	0.062	0.920
			0.7	0.842	0.274	0.234	0.282	0.162	0.842	0.974	0.232	0.220	0.242	0.082	0.920
			0.9	0.820	0.338	0.248	0.386	0.234	0.796	0.856	0.244	0.236	0.300	0.108	0.772
	2	4	0.4	0.980	0.410	0.336	0.446	0.232	0.948	0.998	0.390	0.342	0.424	0.092	0.988
			0.7	0.918	0.494	0.366	0.514	0.304	0.858	0.984	0.500	0.378	0.462	0.124	0.788
			0.9	0.902	0.560	0.418	0.594	0.366	0.886	0.902	0.508	0.388	0.476	0.154	0.670
	4	4	0.4	0.990	0.578	0.416	0.510	0.242	0.856	0.998	0.560	0.398	0.486	0.172	0.822
			0.7	0.956	0.636	0.488	0.564	0.302	0.792	0.990	0.678	0.526	0.582	0.194	0.516
			0.9	0.966	0.820	0.678	0.790	0.484	0.916	0.912	0.834	0.650	0.708	0.300	0.530

The methods compared are the Score and Mixed Model (SM), Longitudinal Partial Credit Model (LPCM), Time to HRQoL score deterioration as compared to the baseline score (TTD baseline) or the best previous score (TTD best) and time until definitive deterioration of the HRQoL score as compared to the baseline score (TUDD baseline) or the best previous score (TUDD best) for different values of sample size (N), items (I), response category per item (J) and correlations between HRQoL measures (ρ).

### Incomplete data

With intermittent missing forms and drop-out, the type I error rate was close to the expected value (5%) for all methods, whatever the proportion of missing data (Table 5 and Table A1 in Additional file 3). The statistical power of the test for an interaction between treatment arm and time (Table 6 and Table A2 in Additional file 3) decreased for the SM method and TTD/TUDD approaches, except for TUDD as compared to the best previous score (“TUDD best”). With 30% missing data as compared to complete case data, 5 measurement times, N = 200 patients, I = 4 items, ρ = 0.7, statistical power decreased from 81% to 76% for SM method, from 55% to 40% for “TTD baseline”, from 39.4% to 28.4% for “TTD best” and from 46% to 39% for “TUDD baseline”.

**Table 5:** **Type I error of the test of interaction between treatment arm and time, for datasets simulated with intermittent missing forms and monotone missing data**

					**5 measures**						**10 measures**					
**N**	**I**	**J**	**ρ**	**π**	**SM**	**TTD baseline**	**TTD best**	**TUDD baseline**	**TUDD best**	**LPCM**	**SM**	**TTD baseline**	**TTD best**	**TUDD baseline**	**TUDD best**	**LPCM**
100	1	4	0.7	0.10	0.068	0.052	0.058	0.058	0.056	0.064	0.052	0.068	0.058	0.066	0.044	0.060
				0.20	0.068	0.042	0.048	0.060	0.078	0.060	0.054	0.048	0.036	0.078	0.050	0.056
				0.30	0.074	0.054	0.050	0.074	0.068	0.064	0.062	0.060	0.050	0.056	0.054	0.056
	2	4	0.7	0.10	0.068	0.052	0.058	0.050	0.050	0.058	0.038	0.048	0.040	0.040	0.052	0.048
				0.20	0.070	0.050	0.054	0.060	0.066	0.068	0.052	0.060	0.060	0.056	0.060	0.052
				0.30	0.072	0.058	0.062	0.066	0.076	0.070	0.068	0.068	0.056	0.056	0.054	0.052
	4	4	0.7	0.10	0.076	0.036	0.048	0.068	0.062	0.078	0.046	0.054	0.072	0.048	0.048	0.048
				0.20	0.074	0.036	0.052	0.066	0.068	0.080	0.076	0.054	0.044	0.070	0.052	0.052
				0.30	0.058	0.058	0.066	0.066	0.054	0.050	0.070	0.058	0.056	0.058	0.046	0.074
200	1	4	0.7	0.10	0.058	0.042	0.038	0.054	0.052	0.058	0.042	0.066	0.058	0.068	0.068	0.056
				0.20	0.076	0.050	0.056	0.046	0.042	0.062	0.052	0.042	0.034	0.052	0.054	0.052
				0.30	0.048	0.052	0.064	0.062	0.046	0.046	0.064	0.058	0.038	0.050	0.044	0.066
	2	4	0.7	0.10	0.046	0.044	0.048	0.038	0.046	0.042	0.044	0.054	0.046	0.052	0.058	0.058
				0.20	0.050	0.056	0.068	0.062	0.058	0.044	0.052	0.054	0.036	0.062	0.044	0.058
				0.30	0.062	0.048	0.040	0.066	0.072	0.060	0.038	0.044	0.038	0.038	0.040	0.044
		7	0.7	0.10	0.044	0.056	0.054	0.056	0.044	0.050	0.036	0.052	0.062	0.050	0.048	0.044
				0.20	0.058	0.056	0.044	0.070	0.056	0.046	0.046	0.058	0.068	0.054	0.048	0.058
				0.30	0.072	0.058	0.060	0.060	0.076	0.062	0.034	0.074	0.058	0.062	0.066	0.046
	4	4	0.7	0.10	0.040	0.040	0.056	0.042	0.044	0.052	0.042	0.056	0.062	0.054	0.056	0.052
				0.20	0.040	0.052	0.052	0.064	0.058	0.054	0.050	0.060	0.036	0.050	0.056	0.062
				0.30	0.054	0.054	0.054	0.056	0.064	0.052	0.042	0.046	0.056	0.044	0.052	0.054
300	1	4	0.7	0.10	0.032	0.054	0.048	0.046	0.038	0.034	0.040	0.066	0.070	0.080	0.060	0.038
				0.20	0.042	0.072	0.058	0.056	0.044	0.044	0.042	0.054	0.046	0.060	0.038	0.050
				0.30	0.046	0.050	0.048	0.048	0.046	0.042	0.046	0.052	0.044	0.064	0.052	0.040
	2	4	0.7	0.10	0.038	0.044	0.042	0.048	0.040	0.042	0.040	0.042	0.050	0.048	0.048	0.046
				0.20	0.052	0.048	0.056	0.042	0.048	0.054	0.044	0.062	0.056	0.044	0.044	0.048
				0.30	0.036	0.052	0.050	0.046	0.044	0.048	0.034	0.036	0.050	0.054	0.064	0.034
	4	4	0.7	0.10	0.034	0.054	0.044	0.048	0.048	0.038	0.034	0.056	0.048	0.044	0.056	0.038
				0.20	0.038	0.054	0.056	0.064	0.054	0.036	0.034	0.042	0.036	0.048	0.050	0.040
				0.30	0.038	0.036	0.038	0.050	0.046	0.040	0.044	0.048	0.038	0.052	0.060	0.05

The methods compared are Score and Mixed Model (SM), longitudinal Partial Credit Model (LPCM), Time to HRQoL score deterioration as compared to the baseline score (TTD baseline) or the best previous score (TTD best) and time until definitive deterioration of the HRQoL score as compared to the baseline score (TUDD baseline) or the best previous score (TUDD best) for different values of sample size (N), items (I), response categories per item (J), correlations between HRQoL measures (ρ) and proportion of missing data (π).

**Table 6:** **Power of the test of interaction between treatment arm and time, for datasets simulated with intermittent missing forms and monotone missing data**

					**5 measures**						**10 measures**					
**N**	**I**	**J**	**ρ**	**π**	**SM**	**TTD baseline**	**TTD best**	**TUDD baseline**	**TUDD best**	**LPCM**	**SM**	**TTD baseline**	**TTD best**	**TUDD baseline**	**TUDD best**	**LPCM**
100	1	4	0.7	0.10	0.396	0.124	0.100	0.140	0.102	0.366	0.568	0.118	0.130	0.108	0.094	0.502
				0.20	0.358	0.098	0.088	0.134	0.104	0.336	0.534	0.100	0.094	0.128	0.058	0.478
				0.30	0.342	0.126	0.092	0.120	0.088	0.318	0.490	0.134	0.098	0.146	0.094	0.430
	2	4	0.7	0.10	0.474	0.224	0.168	0.208	0.144	0.414	0.596	0.170	0.150	0.182	0.054	0.380
				0.20	0.446	0.206	0.164	0.192	0.136	0.402	0.560	0.182	0.136	0.200	0.086	0.400
				0.30	0.404	0.160	0.142	0.162	0.132	0.370	0.506	0.148	0.126	0.192	0.094	0.376
	4	4	0.7	0.10	0.520	0.266	0.214	0.252	0.172	0.380	0.652	0.264	0.210	0.220	0.124	0.258
				0.20	0.514	0.256	0.190	0.248	0.164	0.406	0.626	0.274	0.188	0.262	0.132	0.286
				0.30	0.462	0.232	0.174	0.224	0.160	0.344	0.580	0.216	0.164	0.210	0.116	0.306
200	1	4	0.7	0.10	0.652	0.208	0.160	0.202	0.162	0.630	0.838	0.146	0.168	0.174	0.076	0.774
				0.20	0.644	0.206	0.178	0.218	0.162	0.626	0.810	0.212	0.190	0.216	0.114	0.740
				0.30	0.634	0.222	0.136	0.208	0.146	0.616	0.764	0.156	0.142	0.202	0.108	0.716
	2	4	0.7	0.10	0.754	0.318	0.236	0.332	0.186	0.690	0.902	0.316	0.244	0.280	0.082	0.650
				0.20	0.748	0.346	0.226	0.378	0.218	0.692	0.850	0.352	0.274	0.322	0.116	0.632
				0.30	0.682	0.280	0.212	0.320	0.212	0.644	0.818	0.282	0.204	0.292	0.146	0.678
		7	0.7	0.10	0.816	0.452	0.348	0.456	0.294	0.72	0.92	0.472	0.338	0.412	0.124	0.478
				0.20	0.784	0.448	0.328	0.416	0.278	0.666	0.924	0.424	0.308	0.408	0.154	0.554
				0.30	0.786	0.394	0.254	0.404	0.260	0.71	0.862	0.438	0.29	0.374	0.170	0.616
	4	4	0.7	0.10	0.812	0.470	0.334	0.412	0.248	0.652	0.910	0.462	0.366	0.392	0.156	0.434
				0.20	0.816	0.468	0.316	0.402	0.216	0.686	0.890	0.442	0.314	0.386	0.174	0.482
				0.30	0.762	0.396	0.284	0.390	0.266	0.652	0.862	0.422	0.266	0.412	0.164	0.522
300	1	4	0.7	0.10	0.842	0.320	0.254	0.336	0.212	0.822	0.956	0.202	0.226	0.252	0.114	0.910
				0.20	0.820	0.286	0.224	0.294	0.196	0.800	0.926	0.236	0.208	0.284	0.138	0.888
				0.30	0.756	0.266	0.214	0.278	0.198	0.736	0.910	0.276	0.192	0.290	0.144	0.886
	2	4	0.7	0.10	0.914	0.458	0.322	0.454	0.284	0.858	0.980	0.432	0.328	0.436	0.114	0.802
				0.20	0.890	0.400	0.278	0.434	0.260	0.858	0.964	0.416	0.274	0.418	0.166	0.836
				0.30	0.862	0.416	0.302	0.442	0.262	0.818	0.944	0.450	0.268	0.432	0.196	0.830
	4	4	0.7	0.10	0.964	0.660	0.456	0.618	0.324	0.846	0.992	0.636	0.480	0.538	0.200	0.596
				0.20	0.932	0.626	0.430	0.580	0.338	0.834	0.980	0.606	0.400	0.560	0.236	0.646
				0.30	0.934	0.574	0.386	0.572	0.364	0.856	0.972	0.598	0.376	0.562	0.246	0.724

The methods compared are the Score and Mixed Model (SM), longitudinal Partial Credit Model (LPCM), Time to HRQoL score deterioration as compared to the baseline score (TTD baseline) or the best previous score (TTD best) and time until definitive deterioration of the HRQoL score as compared to the baseline score (TUDD baseline) or the best previous score (TUDD best) for different values of sample size (N), items (I), response categories per item (J), correlations between HRQoL measures (ρ) and proportion of missing data (π).

Regarding TUDD, as compared to the best previous score (“TUDD best”), statistical power generally increased. With 30% missing data as compared to complete case data, with 5 measurement times, N = 300 patients, I = 4 items, ρ = 0.7, the statistical power increased from 30% to 36% for TUDD as compared to the best previous score.

Regarding the LPCM method, the statistical power decreased or remained stable with 5 measurement times, whereas it generally increased for 10 measurement times. With 10 measurement times, N = 300 patients, I = 4 items, ρ = 0.9, the statistical power of LPCM method increased from 53% with complete data to 77% with 30% missing data.

With intermittent missing items and drop-out, results were close to those with intermittent missing forms and drop out. The type I error rate still remained stable and close to the expected value (5%) for all methods, whatever the proportion of missing data generated (see Table A3 in Additional file 3). The statistical power of the test of interaction between treatment arm and time (see Table A4 in Additional file 3) slightly decreased for the SM method and TTD/TUDD approaches, except for TUDD as compared to the best previous score (“TUDD best”), and regardless of the number of measurement times, items, response categories per item ore correlations between HRQoL measures. This trend was generally more pronounced than for intermittent missing forms and drop out. With 30% missing data, 5 measurement times, N = 200 patients, I = 4 items, ρ = 0.7, the statistical power decreased from 81% to 72% for the SM method, from 55% to 28% for “TTD baseline”, from 39% to 20% for “TTD best” and from 46% to 28% for “TUDD baseline”.

The statistical power of the LPCM method increased with intermittent missing data. This trend was generally more pronounced than for intermittent missing forms and drop out. With 10 measurement times, N = 300 patients, I = 4 items, ρ = 0.9, the statistical power of the LPCM method increased from 53% with complete data to 78% with 30% missing data.

**Figure 1:**
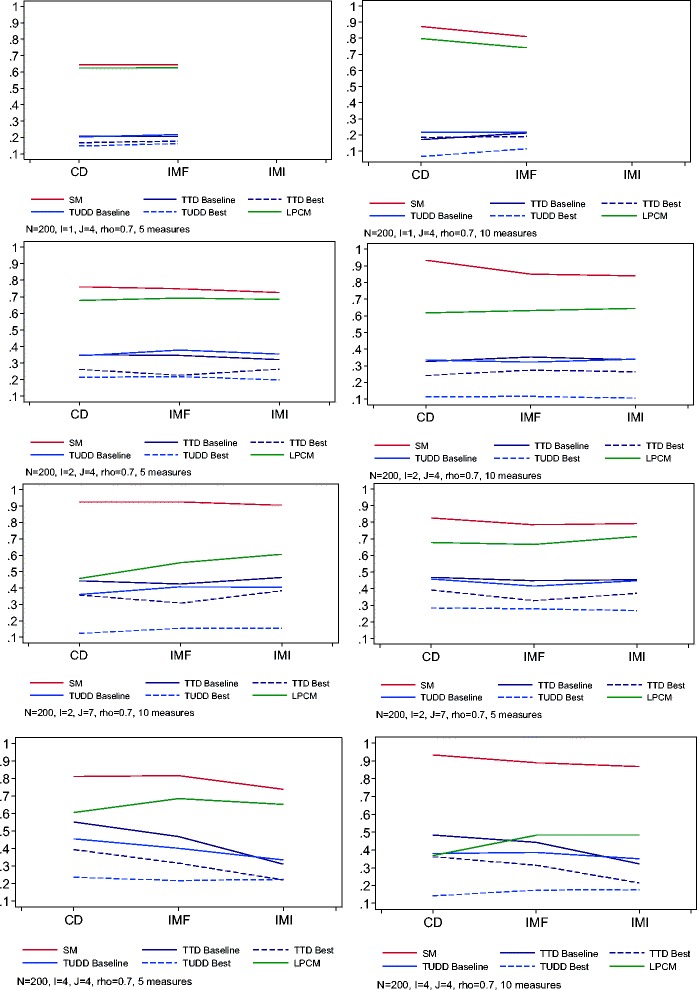
**Power of the test of interaction between treatment arm and time for complete datasets (CD), datasets with intermittent missing forms and monotone missing data (IMF), and datasets with intermittent missing items and monotone missing data (IMI).** The methods compared are the Score and Mixed Model (SM), longitudinal Partial Credit Model (LPCM), Time to HRQoL score deterioration as compared to the baseline score (TTD baseline) or the best previous score (TTD best) and time until definitive deterioration of the HRQoL score as compared to the baseline score (TUDD baseline) or the best previous score (TUDD best) for different values of sample size (N), items (I), correlations between HRQoL measure (ρ) and the proportion of missing data, which was fixed at π = 0.20.

Figure 1 shows the statistical power for all methods with complete data, intermittent missing forms and drop out, and intermittent missing items and drop-out, for N =200 patients, moderate correlation (ρ = 0.7) and 20% missing data. The statistical power of the SM method and TTD/TUDD approach remained stable or decreased for incomplete data as compared to complete data, for I = 2 or 4 items, whatever the number of measurement times, and particularly with intermittent missing item and drop-out. For the same parameter values, the statistical power of the LPCM method increased for incomplete data as compared to complete data. For I = 1 item and 5 measurement times, the statistical power of all methods remained stable. For 10 measurement times, the statistical power decreased in the presence of intermittent missing data for the SM approach, whereas it increased for the TTD/TUDD and LPCM approaches. Finally, this figure confirms that the SM method is the most powerful method, regardless of the scenario considered and the presence or not of missing data.

## Discussion

In order for HRQoL to be recognized as a major endpoint in oncology clinical trials to qualify for the patient the clinical benefit of a new therapeutic strategy, guidelines for longitudinal analyses are required. Three main methods can be proposed to analyze longitudinal HRQoL data, namely the SM method; a time-to-event approach based on the TTD; and the LPCM approach. This study is the first to compare these techniques for longitudinal analysis of HRQoL data, with polytomous items and more than three measurement times. Moreover, our simulation study is the first to address the interaction effect between treatment arm and time in the context of longitudinal HRQoL data, which corresponds to the conditions of randomized clinical trials with no group effect at baseline. Finally, both intermittent and monotone missing data depending on patients’ HRQoL level (MNAR profile) were studied, thereby approaching the actual conditions of clinical trials.

The results obtained on complete data show that the type I error rate was close to the expected value (5%) for all methods. Moreover, the SM model was the most powerful method to highlight an interaction between treatment arm and time. The statistical power of the TTD/TUDD approach, (whatever the definition of deterioration considered), was very low for single-item dimensions, even with a large sample size. This can be explained by the fact that only four possible values exist for the score. Indeed, we suggest that such an approach be avoided for single-item dimensions, meaning that 6 of the 15 dimensions of the QLQ-C30 questionnaire are concerned by this caveat. The statistical power of the LPCM was close to those of the SM model for 5 measurement times, but decreased for scenarios with 10 measurement times. The statistical power of the different methods compared was also influenced by the level of correlation between HRQoL measures (*ρ* parameter). When the correlation increased, the statistical power of the SM and LPCM methods generally decreased, whereas those of the TTD/TUDD approach increased, regardless of the value of the other parameters. The correlation between HRQoL measures was strong if the patient’s HRQoL level at one time could accurately predict his/her level at the next time point. This could reflect closely spaced measures, i.e. some intensive HRQoL measures, as for clinical trials where there is rapid change in the patient’s health status. Conversely, a weak correlation between HRQoL measures could correspond to more distant measures, reflecting a cohort study design.

With intermittent missing data (missing items or missing forms) and drop-out, the type I error rate remained closed to the expected value for all statistical methods, whatever the proportion of missing data and the scenario considered. The statistical power generally decreased for the SM and TTD/TUDD approaches, except for TUDD as compared to the best previous score. For this definition, the statistical power generally increased or remained stable with the simulation of missing data. This could be explained by the simulation of missing data depending on HRQoL level, i.e. patients with a low HRQoL level were more likely to present missing data. Indeed, an improvement of HRQoL level was more likely to be observed (no missing data) than a deterioration, and this improvement would represent the new reference score for “TUDD best”. Thus, a small deterioration of at least 5 points compared to this new reference score was not considered as a deterioration as compared to the baseline score. Finally, this deterioration was more likely to be followed by monotone missing data, involving a definitive deterioration as compared to the best previous score.

The same trends were observed for all methods regarding statistical power, whatever the type of missing data considered (intermittent missing items or missing forms). However, the statistical power decreased more for the SM and TTD/TUDD approaches in the presence of intermittent missing items than when there were intermittent missing forms. For analyses with intermittent missing items, the score could be estimated if at least 50% of the items had been answered, and on the assumption that missing items are not informative of the patient’s HRQoL level. This could result in an overestimation or underestimation of the patient’s HRQoL level, which could induce a bias in the longitudinal analysis.

As highlighted in other studies [14,15], these results emphasize the limitations of the personal mean score imputation method, despite the fact that it is the most commonly used technique for computing scores. Indeed, it should be avoided, particularly when the proportion of missing data is high. Regarding the LPCM method, the statistical power increased more in the presence of intermittent missing items than when there were intermittent missing forms. This is due to the specific objectivity property of Rasch-family models, which can highly accurately estimate the latent trait (i.e. HRQoL), even with few items answered [12]. This is because a minimum information is provided (at least one item is answered), whereas with missing forms, no information is available for IRT models. Moreover, it seems that the LPCM is more powerful with few measurement time points, since the LPCM has greater power with 5 than with 10 measurement times. Thus, when missing data were generated, the statistical power of LPCM increased.

Previous studies comparing score-based approaches with a Rasch-based approach have highlighted the similar performance of the SM and longitudinal Rasch models in case of complete data [13] and in the presence of monotone missing data [14]. These studies also showed that Rasch-family models seem to be more efficient than SM models in the presence of informative intermittent missing data [15]. In our study, we also highlight that the statistical power of the IRT models was less affected by the presence of missing data than those of the SM method. However, contrary to previous published studies, the SM method was generally more powerful in our study than the IRT model for both complete and incomplete data with informative missing data, and particularly with 10 measurement times. The good results of the SM model could be explained by a bias from fixed effect estimations, since there are several data characteristics that the SM model does not take into account, such as the ceiling and floor effects, or asymmetric data [34,35]. It is also important to note that the SM method generally required the normality of the score studied, which cannot be respected for single-item scales of the QLQ-C30 questionnaire with only 4 possible values for the scores. These discrepancies with the literature may also be partly due to the number of measurement times considered. The IRT models seem to be less powerful when the number of measurement times is high. Moreover, in previous studies, researchers chose to proceed in two steps to construct the longitudinal IRT model, namely estimation of the item parameters and HRQoL latent trait for each person at each time in a first step, and then modeling of the link between the latent trait and the time using a linear mixed model. Our design integrated at least five measures, thereby reflecting a longitudinal design, similar to that used in clinical trials. Moreover, polytomous items were used in our research, whereas dichotomous items were used in previous studies. Finally, we investigated the interaction between treatment arm and time in our study, whereas previous studies analyzed only the time effect [13-15] or the group effect [16]. It therefore appears crucial to pursue research in this area to test the ability of these models in the context of polytomous items.

In our study, both linear and non-linear mixed models and time to event analysis were compared. The time to event (i.e. “survival”) approach based on the time to HRQoL score deterioration is relevant in the event of a quicker alteration of patients’ HRQoL in one treatment arm as compared to the other, and if this difference is maintained over time (risk proportionality). Therefore, the absence of an arm effect at baseline is coherent.

Our results correspond to a particular situation - nearly ideal but theoretical - considering that items were derived from an IRT model and that the corresponding symptomatic scale followed a multivariate normal distribution with an auto-regressive covariance matrix. It is necessary to simulate the data using an IRT model in order to guarantee that the fundamental assumptions of the model are respected, as recommended by Holland et al. [32]. Since the parameters of the IRT model are re-estimated, it cannot necessarily bias the results in favor of the IRT model. Nevertheless, it corresponds to an ideal situation that does not reflect real data when the HRQoL questionnaire does not respect an IRT model. Therefore, additional work is in progress to compare these methods on real data collected from several clinical trials with various therapeutic settings, cancer sites and designs. This comparison is mandatory for the validation of the results obtained in the present simulation study.

Each data set is different, and routinely using same statistical analyses must be prevented in order to retain an open and critical view. However, standardization of longitudinal analysis of HRQoL data in oncology clinical trials is essential in order to allow proper comparison of results between trials. For example, two recent phase III clinical trials investigating the impact of adding bevacizumab to standard therapy in newly diagnosed glioblastoma, applied two different approaches (SM and TUDD) to analyze longitudinal HRQoL data. The results are divergent and compromise conclusions about the clinical value of adding bevacizumab, since overall survival was not improved [36,37]. To date, results from HRQoL studies have not been salient enough to lead to changes in clinical practice. It is also necessary to provide decision-makers with results that are clinical meaningful and easy to understand [38]. In this context, the TTD/TUDD approach is attractive for clinicians, because it is based on Kaplan–Meier survival curves and hazard ratios to qualify effect size, as with other well-known and important time-to-event outcomes in oncology (e.g. overall survival or progression-free survival). However, this approach should be used with caution in light of our results. Moreover, as already shown for progression-free survival [39], the time interval between assessments of HRQoL could influence the Kaplan Meier estimation, thus resulting in an overestimation of TUDD. Since the true time when HRQoL deteriorates may be unknown, dedicated statistic approaches dealing with interval assessment may be proposed. It also seems essential to properly study the profile of missing data in advance, so as to propose a suitable method of score imputation in case of intermittent missing items with an MNAR profile. Some methods have to be developed for use in conjunction with the TTD, such as pattern mixture models for SM model [40], in order to take into account missing data with an MNAR profile. Survival analysis, such as the time to HRQoL score deterioration, only gives unbiased results when censoring is independent of the event. In oncology clinical trials, patients who have a very low HRQoL level are more likely to drop out, and thus the censure could be dependent on the event deterioration. In this case, sensitivity analysis should be performed considering patients who dropped out before the planned end of the study as an event.

All-cause death is usually taken into account as an event, particularly in an advanced setting [18]. However, death was not integrated into our simulation algorithm, which may explain in part the low statistical power of the TTD approach. Moreover, one advantage of this method compared to the mixed models is its adaptability to different therapeutic settings (adjuvant or advanced settings) with consideration of a transient or definitive deterioration, and with or without integration of death as an event.

In conclusion, the SM model was clearly the most effective method, although the nature of the raw data in the questionnaire means that the application of SM models in this context remains open to criticism. The TTD/TUDD approach, which is often used in the longitudinal analysis of HRQoL in oncology, should be used with caution on single-item dimensions of the EORTC questionnaires. Finally, while the LPCM was more adapted to this type of data, it was ultimately difficult to implement and less efficient than the SM model.

## Additional files

Additional file 1:
**Formulae of the Score and Mixed Model and Longitudinal Partial Credit model.**
Additional file 2:
**Generation of missing data.**
Additional file 3:
**Complementary results obtained with intermittent missing items and monotone missing data.**

## Abbreviations

CTT: Classical test theory
EORTC: European Organization for Research and Treatment of Cancer
HRQoL: Health-related quality of life
IRT: Item response theory
LPCM: Longitudinal partial credit model
MAR: Missing at random
MCAR: Missing completely at random
MCID: Minimal clinically important difference
MNAR: Missing not at random
PCM: Partial credit model
SM: Score and mixed model
TTD: Time to deterioration
TUDD: Time until definitive deterioration
