# Supplementary material for: Comparison of three longitudinal analysis models for the health-related quality of life in oncology: a simulation study
Source: Health Qual Life Outcomes. 2014 Dec 31;12:192. doi: 10.1186/s12955-014-0192-2 (PMC4326524; doi:10.1186/s12955-014-0192-2)
Supplement: Additional file 1: — Formulae of the Score and Mixed Model and Longitudinal Partial Credit model. [file 12955_2014_192_MOESM1_ESM.docx]

**Additional File 1 – Formulas of the Score and Mixed Model and Longitudinal Partial Credit model**

**A - *Score and mixed model***

The SM model considered can be written as follows:

$$Y_{n}^{(t)}=c+\nu*{arm}_{n}*t+ \gamma*t+ u_{0,n}+u_{1,n}*t+ \varepsilon_{n}^{\left( t \right)}$$

$$\left( u_{0,n},u_{1,n} \right)\sim N\left( \binom{0}{0},\Sigma\right)$$

and $\varepsilon_{n}^{\left( t \right)} \sim N\left( 0,\sigma^{2} \right)$ independent;

Where:

- $Y_{n}^{(t)}$ is the score of the patient $n$ at time $t$,
- $c$ is a constant,
- ${arm}_{n}$ is the treatment arm of patient $n$ (equal to 0 or 1),
- $\nu$ is a fixed interaction effect between treatment arm and time,
- $\gamma$ is a fixed time effect,
- $u_{0,n}$ is a random effect on patient n,
- $u_{1,n}$ is a random time effect on patient n,
- $\Sigma$ is the variance matrix of random effects$\left( u_{0,n},u_{1,n} \right)$,
- $\varepsilon_{n}^{\left( t \right)}$ is the residual of patient n at time t,
- $\sigma^{2}$ is the residual variance.

**B - *Longitudinal mixed partial credit model***

The Partial Credit Model models the probability for one individual $n$ to choose the response category $k$ among the $m_{j}$ possible responses for the item $j$ (i.e. generalized linear mixed model with a multinomial logit link function) given the latent trait $\theta_{n}$ and the category difficulty parameters $\delta_{j,1},\ldots,\delta_{j,m_{j}}$ for the item :

$$P\left( X_{n,j}=k | \theta_{n}, \delta_{j,1},\ldots,\delta_{j,m_{j}} \right)= \frac{\exp(k\theta_{n} -\sum_{i=1}^{k} \delta_{j,i})}{\sum_{h=1}^{m_{j}} \exp(h\theta_{n} -\sum_{i=1}^{h} \delta_{j,i})}$$

As all Rasch-family models, the PCM relies on three fundamental assumptions:

1. the unidimensionality of the latent trait, i.e. all considered items must measure the same concept or the same HRQoL dimension for example,
2. the monotonicity , i.e. the probability to choose the response category $k$ or a higher response category is increasing in function of the latent trait,
3. and the local independence of the items conditionally to the latent trait: i.e. the items answers must be independent from each other given the latent trait.

The Longitudinal Partial Credit Model that we considered can be written as follows:

$$P\left( X_{n,j}=k | {\theta_{n}}^{(t)}, \delta_{j,1},\ldots,\delta_{j,m_{j}} \right)= \frac{\exp(k{\theta_{n}}^{(t)} -\sum_{i=1}^{k} \delta_{j,i})}{\sum_{h=1}^{m_{j}} \exp(h{\theta_{n}}^{(t)} -\sum_{i=1}^{h} \delta_{j,i})}$$

$${\theta_{n}}^{(t)}=\nu*{arm}_{n}*t+ \gamma*t+ u_{0,n}+u_{1,n}*t$$

$$\left( u_{0,n},u_{1,n} \right)\sim N\left( \binom{0}{0},\Sigma\right)$$

where the latent trait is decomposed linearly with the same effects, as for the SM.
