# Supplementary material for: Comparison of three longitudinal analysis models for the health-related quality of life in oncology: a simulation study
Source: Health Qual Life Outcomes. 2014 Dec 31;12:192. doi: 10.1186/s12955-014-0192-2 (PMC4326524; doi:10.1186/s12955-014-0192-2)
Supplement: Additional file 2: — Generation of missing data. [file 12955_2014_192_MOESM2_ESM.docx]

**Additional File 2 – Generation of missing data**

*Generation of missing data*

A latent variable $\varphi$, defined as the missing data propensity, was used to simulate missing data [[32](#_ENREF_32)].

$\varphi$ followed a multinormal distribution with mean (0 0 0 0 0)’ and a variance covariance matrix equal to $\left( \begin{matrix} \begin{matrix} \begin{matrix} 1 & {\rho^{2}}_{\theta\varphi}\rho\\ {\rho^{2}}_{\theta\varphi}\rho& 1 \\ {\rho^{2}}_{\theta\varphi}\rho^{2} & {\rho^{2}}_{\theta\varphi}\rho\end{matrix} & \begin{matrix} {\rho^{2}}_{\theta\varphi}\rho^{2} & {\rho^{2}}_{\theta\varphi}\rho^{3} & {\rho^{2}}_{\theta\varphi}\rho^{4} \\ {\rho^{2}}_{\theta\varphi}\rho& {\rho^{2}}_{\theta\varphi}\rho^{2} & {\rho^{2}}_{\theta\varphi}\rho^{3} \\ 1 & {\rho^{2}}_{\theta\varphi}\rho& {\rho^{2}}_{\theta\varphi}\rho^{2} \end{matrix} \end{matrix} \\ \begin{matrix} \begin{matrix} {\rho^{2}}_{\theta\varphi}\rho^{3} & {\rho^{2}}_{\theta\varphi}\rho^{2} \\ {\rho^{2}}_{\theta\varphi}\rho^{4} & {\rho^{2}}_{\theta\varphi}\rho^{3} \end{matrix} & \begin{matrix} {\rho^{2}}_{\theta\varphi}\rho& 1 & {\rho^{2}}_{\theta\varphi}\rho\\ {\rho^{2}}_{\theta\varphi}\rho^{2} & {\rho^{2}}_{\theta\varphi}\rho& 1 \end{matrix} \end{matrix} \end{matrix} \right)$ for 5 measures.

$\rho_{\theta\varphi}$ represented the correlation between the latent trait $\theta$ (the HRQoL level) and the latent variable $\varphi$. The probability for a patient $i$ to present a missing item at time $t$ depended on his missing data propensity and is defined as:

$$p_{i,t}=P\left( {{MD}_{i}}^{\left( t \right)}=1 \right|{\varphi_{i}}^{(t)}, \pi_{min}^{(t)}, \pi_{max}^{(t)})= \pi_{min}^{(t)}+\left( \pi_{max}^{\left( t \right)}- \pi_{min}^{\left( t \right)} \right)\frac{exp({\varphi_{i}}^{(t)})}{1+ exp({\varphi_{i}}^{(t)})}$$

with ${{MD}_{i}}^{\left( t \right)}=1$ if patient $i$ presented a missing data at time $t$. $\pi_{min}^{(t)}$ and $\pi_{max}^{(t)}$ were defined respectively as the minimum and maximum individual probability to present a missing data at time $t$. The expected proportion of missing data then equal to $\pi^{(t)}= \frac{\pi_{min}^{\left( t \right)}+ \pi_{max}^{\left( t \right)}}{2}$. We fixed $\pi_{min}^{\left( t \right)}=0.01$ and $\pi_{max}^{\left( t \right)}=2 \pi^{(t)}-0.01$.

Patient $i$ presented a missing data at time $t$ according to a Bernoulli distribution with $p_{i,t}$ parameter.

Only simulation of a MNAR profile was performed. Indeed, only the MNAR profile is informative and can increase the risk of bias in the longitudinal analysis. Patients with a low HRQoL level or a high symptomatic level were supposed to be more likely to present missing data. As $\theta$ represented a symptomatic HRQoL dimension, $\rho_{\theta\varphi}>0$ because a high level for the latent trait $\theta$ represented a high symptomatic level. We fixed $\rho_{\theta\varphi}=0.7$ to simulate a moderate informative MNAR profile.

Two types of intermittent missing data were considered: intermittent missing forms and intermittent missing items. Regarding intermittent missing forms, simulation of missing data was performed at each measurement time: if patient $i$ presents a missing data at time$t$, then all items of the dimension are missing for that patient at time $t$. For intermittent missing item, a Bernoulli distribution with $p_{i,t}$ parameter was simulated for each item.
