# Supplementary material for: Comparison of three longitudinal analysis models for the health-related quality of life in oncology: a simulation study
Source: Health Qual Life Outcomes. 2014 Dec 31;12:192. doi: 10.1186/s12955-014-0192-2 (PMC4326524; doi:10.1186/s12955-014-0192-2)
Supplement: Additional file 3: — Complementary results obtained with intermittent missing items and monotone missing data. [file 12955_2014_192_MOESM3_ESM.doc]

# Additional file 3 – Complementary results obtained with intermittent missing items and monotone missing data

**Table A1 - Type I error of the test of interaction between treatment arm and time for datasets simulated with intermittent missing forms and monotone missing data**

|  |  |  |  |  | 5 measures | | | | | | 10 measures | | | | | |
| --- | --- | --- | --- | --- | --- | --- | --- | --- | --- | --- | --- | --- | --- | --- | --- | --- |
| N | I | J | ρ | π | SM | TTD baseline | TTD best | TUDD baseline | TUDD best | LPCM | SM | TTD baseline | TTD best | TUDD baseline | TUDD best | LPCM |
| 100 | 1 | 4 | 0.4 | 0.10 | 0.058 | 0.074 | 0.064 | 0.070 | 0.068 | 0.060 | 0.048 | 0.068 | 0.100 | 0.086 | 0.064 | 0.130 |
|  |  |  |  | 0.20 | 0.076 | 0.052 | 0.058 | 0.054 | 0.048 | 0.064 | 0.064 | 0.060 | 0.048 | 0.054 | 0.052 | 0.126 |
|  |  |  |  | 0.30 | 0.068 | 0.070 | 0.068 | 0.062 | 0.066 | 0.066 | 0.052 | 0.056 | 0.056 | 0.048 | 0.054 | 0.082 |
|  |  |  | 0.9 | 0.10 | 0.078 | 0.048 | 0.042 | 0.054 | 0.052 | 0.082 | 0.040 | 0.052 | 0.048 | 0.042 | 0.064 | 0.036 |
|  |  |  |  | 0.20 | 0.056 | 0.050 | 0.038 | 0.052 | 0.044 | 0.046 | 0.066 | 0.042 | 0.046 | 0.054 | 0.042 | 0.074 |
|  |  |  |  | 0.30 | 0.044 | 0.038 | 0.052 | 0.044 | 0.062 | 0.038 | 0.060 | 0.048 | 0.046 | 0.040 | 0.052 | 0.052 |
|  | 2 | 4 | 0.4 | 0.10 | 0.060 | 0.076 | 0.070 | 0.064 | 0.066 | 0.066 | 0.042 | 0.048 | 0.070 | 0.060 | 0.052 | 0.054 |
|  |  |  |  | 0.20 | 0.064 | 0.040 | 0.048 | 0.058 | 0.064 | 0.066 | 0.042 | 0.068 | 0.048 | 0.060 | 0.056 | 0.042 |
|  |  |  |  | 0.30 | 0.054 | 0.044 | 0.038 | 0.056 | 0.058 | 0.066 | 0.058 | 0.054 | 0.076 | 0.056 | 0.060 | 0.056 |
|  |  |  | 0.9 | 0.10 | 0.055 | 0.056 | 0.064 | 0.056 | 0.058 | 0.050 | 0.070 | 0.060 | 0.058 | 0.062 | 0.058 | 0.052 |
|  |  |  |  | 0.20 | 0.066 | 0.058 | 0.048 | 0.052 | 0.054 | 0.060 | 0.050 | 0.042 | 0.054 | 0.040 | 0.054 | 0.056 |
|  |  |  |  | 0.30 | 0.064 | 0.046 | 0.070 | 0.062 | 0.072 | 0.066 | 0.060 | 0.052 | 0.044 | 0.050 | 0.052 | 0.046 |
|  | 4 | 4 | 0.4 | 0.10 | 0.074 | 0.068 | 0.076 | 0.070 | 0.060 | 0.056 | 0.064 | 0.050 | 0.058 | 0.056 | 0.050 | 0.050 |
|  |  |  |  | 0.20 | 0.074 | 0.050 | 0.052 | 0.078 | 0.052 | 0.058 | 0.048 | 0.060 | 0.058 | 0.058 | 0.058 | 0.030 |
|  |  |  |  | 0.30 | 0.066 | 0.060 | 0.062 | 0.066 | 0.086 | 0.066 | 0.064 | 0.076 | 0.068 | 0.05 | 0.058 | 0.058 |
|  |  |  | 0.9 | 0.10 | 0.050 | 0.060 | 0.082 | 0.046 | 0.062 | 0.046 | 0.046 | 0.066 | 0.054 | 0.048 | 0.050 | 0.062 |
|  |  |  |  | 0.20 | 0.058 | 0.070 | 0.052 | 0.054 | 0.060 | 0.066 | 0.046 | 0.042 | 0.058 | 0.056 | 0.052 | 0.052 |
|  |  |  |  | 0.30 | 0.058 | 0.062 | 0.042 | 0.066 | 0.066 | 0.058 | 0.034 | 0.034 | 0.054 | 0.064 | 0.072 | 0.068 |
| 200 | 1 | 4 | 0.4 | 0.10 | 0.042 | 0.042 | 0.046 | 0.056 | 0.06 | 0.046 | 0.058 | 0.068 | 0.068 | 0.062 | 0.074 | 0.156 |
|  |  |  |  | 0.20 | 0.056 | 0.052 | 0.058 | 0.046 | 0.044 | 0.060 | 0.038 | 0.044 | 0.046 | 0.054 | 0.042 | 0.122 |
|  |  |  |  | 0.30 | 0.064 | 0.072 | 0.078 | 0.052 | 0.068 | 0.054 | 0.046 | 0.062 | 0.050 | 0.080 | 0.060 | 0.102 |
|  |  |  | 0.9 | 0.10 | 0.050 | 0.072 | 0.06 | 0.052 | 0.054 | 0.050 | 0.032 | 0.054 | 0.036 | 0.060 | 0.052 | 0.036 |
|  |  |  |  | 0.20 | 0.044 | 0.032 | 0.044 | 0.038 | 0.052 | 0.034 | 0.062 | 0.064 | 0.060 | 0.050 | 0.056 | 0.058 |
|  |  |  |  | 0.30 | 0.062 | 0.054 | 0.056 | 0.036 | 0.054 | 0.056 | 0.028 | 0.042 | 0.058 | 0.050 | 0.044 | 0.030 |
|  | 2 | 4 | 0.4 | 0.10 | 0.042 | 0.076 | 0.080 | 0.048 | 0.066 | 0.044 | 0.046 | 0.040 | 0.050 | 0.054 | 0.054 | 0.036 |
|  |  |  |  | 0.20 | 0.048 | 0.060 | 0.070 | 0.056 | 0.056 | 0.052 | 0.046 | 0.034 | 0.056 | 0.056 | 0.064 | 0.058 |
|  |  |  |  | 0.30 | 0.032 | 0.048 | 0.036 | 0.050 | 0.056 | 0.042 | 0.052 | 0.062 | 0.052 | 0.050 | 0.050 | 0.048 |
|  |  |  | 0.9 | 0.10 | 0.050 | 0.042 | 0.048 | 0.052 | 0.032 | 0.052 | 0.046 | 0.050 | 0.060 | 0.054 | 0.054 | 0.046 |
|  |  |  |  | 0.20 | 0.058 | 0.050 | 0.050 | 0.058 | 0.048 | 0.060 | 0.036 | 0.058 | 0.052 | 0.048 | 0.050 | 0.040 |
|  |  |  |  | 0.30 | 0.054 | 0.060 | 0.046 | 0.052 | 0.044 | 0.060 | 0.056 | 0.056 | 0.048 | 0.068 | 0.054 | 0.050 |
|  |  | 7 | 0.4 | 0.10 | 0.052 | 0.056 | 0.072 | 0.05 | 0.044 | 0.054 | 0.046 | 0.062 | 0.040 | 0.060 | 0.054 | 0.046 |
|  |  |  |  | 0.20 | 0.052 | 0.060 | 0.064 | 0.044 | 0.064 | 0.052 | 0.040 | 0.048 | 0.022 | 0.044 | 0.050 | 0.056 |
|  |  |  |  | 0.30 | 0.046 | 0.068 | 0.08 | 0.058 | 0.062 | 0.048 | 0.046 | 0.056 | 0.048 | 0.054 | 0.066 | 0.058 |
|  |  |  | 0.9 | 0.10 | 0.054 | 0.066 | 0.066 | 0.044 | 0.042 | 0.060 | 0.044 | 0.040 | 0.040 | 0.044 | 0.036 | 0.058 |
|  |  |  |  | 0.20 | 0.048 | 0.072 | 0.048 | 0.048 | 0.070 | 0.048 | 0.048 | 0.062 | 0.050 | 0.046 | 0.046 | 0.056 |
|  |  |  |  | 0.30 | 0.060 | 0.072 | 0.054 | 0.082 | 0.072 | 0.068 | 0.056 | 0.052 | 0.044 | 0.066 | 0.062 | 0.044 |
|  | 4 | 4 | 0.4 | 0.10 | 0.048 | 0.068 | 0.068 | 0.056 | 0.06 | 0.056 | 0.054 | 0.058 | 0.060 | 0.052 | 0.06 | 0.056 |
|  |  |  |  | 0.20 | 0.050 | 0.062 | 0.052 | 0.064 | 0.052 | 0.052 | 0.036 | 0.044 | 0.058 | 0.048 | 0.038 | 0.040 |
|  |  |  |  | 0.30 | 0.070 | 0.060 | 0.048 | 0.062 | 0.060 | 0.064 | 0.058 | 0.05 | 0.044 | 0.062 | 0.052 | 0.050 |
|  |  |  | 0.9 | 0.10 | 0.060 | 0.062 | 0.058 | 0.06 | 0.054 | 0.052 | 0.038 | 0.054 | 0.048 | 0.048 | 0.030 | 0.040 |
|  |  |  |  | 0.20 | 0.068 | 0.044 | 0.048 | 0.048 | 0.050 | 0.064 | 0.038 | 0.052 | 0.054 | 0.050 | 0.066 | 0.038 |
|  |  |  |  | 0.30 | 0.054 | 0.046 | 0.050 | 0.056 | 0.054 | 0.044 | 0.052 | 0.058 | 0.068 | 0.042 | 0.050 | 0.048 |
| 300 | 1 | 4 | 0.4 | 0.10 | 0.052 | 0.044 | 0.048 | 0.054 | 0.052 | 0.060 | 0.050 | 0.050 | 0.042 | 0.054 | 0.036 | 0.110 |
|  |  |  |  | 0.20 | 0.052 | 0.050 | 0.036 | 0.048 | 0.040 | 0.052 | 0.030 | 0.042 | 0.052 | 0.054 | 0.058 | 0.092 |
|  |  |  |  | 0.30 | 0.040 | 0.058 | 0.054 | 0.066 | 0.072 | 0.042 | 0.056 | 0.040 | 0.036 | 0.030 | 0.046 | 0.104 |
|  |  |  | 0.9 | 0.10 | 0.048 | 0.058 | 0.040 | 0.044 | 0.036 | 0.052 | 0.056 | 0.048 | 0.046 | 0.044 | 0.074 | 0.058 |
|  |  |  |  | 0.20 | 0.050 | 0.044 | 0.048 | 0.032 | 0.038 | 0.042 | 0.048 | 0.048 | 0.038 | 0.050 | 0.028 | 0.056 |
|  |  |  |  | 0.30 | 0.054 | 0.040 | 0.050 | 0.040 | 0.040 | 0.048 | 0.048 | 0.046 | 0.048 | 0.040 | 0.052 | 0.048 |
|  | 2 | 4 | 0.4 | 0.10 | 0.042 | 0.052 | 0.050 | 0.058 | 0.058 | 0.050 | 0.038 | 0.056 | 0.058 | 0.052 | 0.056 | 0.042 |
|  |  |  |  | 0.20 | 0.046 | 0.040 | 0.046 | 0.060 | 0.050 | 0.048 | 0.058 | 0.060 | 0.038 | 0.042 | 0.042 | 0.062 |
|  |  |  |  | 0.30 | 0.038 | 0.036 | 0.046 | 0.046 | 0.044 | 0.042 | 0.050 | 0.056 | 0.058 | 0.068 | 0.056 | 0.050 |
|  |  |  | 0.9 | 0.10 | 0.074 | 0.062 | 0.042 | 0.064 | 0.058 | 0.072 | 0.044 | 0.054 | 0.066 | 0.056 | 0.058 | 0.050 |
|  |  |  |  | 0.20 | 0.042 | 0.060 | 0.052 | 0.046 | 0.044 | 0.052 | 0.042 | 0.048 | 0.066 | 0.038 | 0.040 | 0.030 |
|  |  |  |  | 0.30 | 0.066 | 0.034 | 0.044 | 0.034 | 0.042 | 0.074 | 0.042 | 0.034 | 0.040 | 0.024 | 0.046 | 0.030 |
|  | 4 | 4 | 0.4 | 0.10 | 0.042 | 0.058 | 0.058 | 0.066 | 0.056 | 0.058 | 0.046 | 0.066 | 0.042 | 0.050 | 0.058 | 0.056 |
|  |  |  |  | 0.20 | 0.050 | 0.050 | 0.056 | 0.064 | 0.042 | 0.054 | 0.036 | 0.052 | 0.048 | 0.056 | 0.046 | 0.040 |
|  |  |  |  | 0.30 | 0.050 | 0.060 | 0.034 | 0.044 | 0.038 | 0.038 | 0.038 | 0.042 | 0.036 | 0.068 | 0.05 | 0.048 |
|  |  |  | 0.9 | 0.10 | 0.038 | 0.048 | 0.048 | 0.044 | 0.060 | 0.048 | 0.038 | 0.050 | 0.032 | 0.046 | 0.048 | 0.036 |
|  |  |  |  | 0.20 | 0.044 | 0.054 | 0.042 | 0.038 | 0.038 | 0.040 | 0.040 | 0.036 | 0.028 | 0.040 | 0.062 | 0.048 |
|  |  |  |  | 0.30 | 0.048 | 0.058 | 0.048 | 0.054 | 0.050 | 0.044 | 0.038 | 0.046 | 0.048 | 0.052 | 0.050 | 0.036 |

Methods compared are the Score and Mixed Model (SM), longitudinal Partial Credit Model (LPCM), Time to HRQoL score deterioration as compared to the baseline score (TTD baseline) or the best previous score (TTD best) and time until definitive deterioration of the HRQoL score as compared to the baseline score (TUDD baseline) or the best previous score (TUDD best) for different values of sample size (N), items (I), response category per item (J), correlations between HRQoL measure (ρ) and proportion of missing data (π).

**Table A2 -** Power of the test of interaction between treatment arm and time for datasets simulated with intermittent missing forms and monotone missing data

|  |  |  |  |  | 5 measures | | | | | | 10 measures | | | | | |
| --- | --- | --- | --- | --- | --- | --- | --- | --- | --- | --- | --- | --- | --- | --- | --- | --- |
| N | I | J | ρ | π | SM | TTD baseline | TTD best | TUDD baseline | TUDD best | LPCM | SM | TTD baseline | TTD best | TUDD baseline | TUDD best | LPCM |
| 100 | 1 | 4 | 0.4 | 0.10 | 0.486 | 0.136 | 0.118 | 0.144 | 0.094 | 0.448 | 0.732 | 0.086 | 0.112 | 0.110 | 0.052 | 0.614 |
|  |  |  |  | 0.20 | 0.434 | 0.120 | 0.098 | 0.134 | 0.090 | 0.376 | 0.666 | 0.100 | 0.104 | 0.136 | 0.094 | 0.610 |
|  |  |  |  | 0.30 | 0.356 | 0.112 | 0.100 | 0.108 | 0.078 | 0.306 | 0.610 | 0.114 | 0.104 | 0.124 | 0.076 | 0.528 |
|  |  |  | 0.7 | 0.10 | 0.396 | 0.124 | 0.100 | 0.140 | 0.102 | 0.366 | 0.568 | 0.118 | 0.130 | 0.108 | 0.094 | 0.502 |
|  |  |  |  | 0.20 | 0.358 | 0.098 | 0.088 | 0.134 | 0.104 | 0.336 | 0.534 | 0.100 | 0.094 | 0.128 | 0.058 | 0.478 |
|  |  |  |  | 0.30 | 0.342 | 0.126 | 0.092 | 0.120 | 0.088 | 0.318 | 0.490 | 0.134 | 0.098 | 0.146 | 0.094 | 0.430 |
|  |  |  | 0.9 | 0.10 | 0.372 | 0.134 | 0.122 | 0.118 | 0.114 | 0.336 | 0.374 | 0.128 | 0.102 | 0.146 | 0.086 | 0.334 |
|  |  |  |  | 0.20 | 0.366 | 0.124 | 0.136 | 0.138 | 0.134 | 0.342 | 0.394 | 0.148 | 0.098 | 0.146 | 0.070 | 0.352 |
|  |  |  |  | 0.30 | 0.338 | 0.138 | 0.112 | 0.150 | 0.090 | 0.308 | 0.384 | 0.136 | 0.108 | 0.138 | 0.090 | 0.340 |
|  | 2 | 4 | 0.4 | 0.10 | 0.536 | 0.170 | 0.146 | 0.190 | 0.120 | 0.492 | 0.802 | 0.150 | 0.134 | 0.196 | 0.076 | 0.684 |
|  |  |  |  | 0.20 | 0.516 | 0.176 | 0.110 | 0.190 | 0.130 | 0.452 | 0.766 | 0.156 | 0.150 | 0.154 | 0.080 | 0.647 |
|  |  |  |  | 0.30 | 0.502 | 0.168 | 0.132 | 0.200 | 0.164 | 0.452 | 0.712 | 0.178 | 0.140 | 0.168 | 0.090 | 0.606 |
|  |  |  | 0.7 | 0.10 | 0.474 | 0.224 | 0.168 | 0.208 | 0.144 | 0.414 | 0.596 | 0.170 | 0.150 | 0.182 | 0.054 | 0.380 |
|  |  |  |  | 0.20 | 0.446 | 0.206 | 0.164 | 0.192 | 0.136 | 0.402 | 0.560 | 0.182 | 0.136 | 0.200 | 0.086 | 0.400 |
|  |  |  |  | 0.30 | 0.404 | 0.160 | 0.142 | 0.162 | 0.132 | 0.370 | 0.506 | 0.148 | 0.126 | 0.192 | 0.094 | 0.376 |
|  |  |  | 0.9 | 0.10 | 0.438 | 0.246 | 0.198 | 0.270 | 0.174 | 0.428 | 0.436 | 0.206 | 0.164 | 0.202 | 0.102 | 0.312 |
|  |  |  |  | 0.20 | 0.438 | 0.218 | 0.168 | 0.234 | 0.172 | 0.424 | 0.424 | 0.218 | 0.162 | 0.218 | 0.118 | 0.326 |
|  |  |  |  | 0.30 | 0.432 | 0.200 | 0.140 | 0.216 | 0.164 | 0.402 | 0.462 | 0.226 | 0.166 | 0.220 | 0.126 | 0.372 |
|  | 4 | 4 | 0.4 | 0.10 | 0.632 | 0.212 | 0.160 | 0.204 | 0.142 | 0.422 | 0.856 | 0.216 | 0.172 | 0.212 | 0.112 | 0.404 |
|  |  |  |  | 0.20 | 0.622 | 0.182 | 0.150 | 0.206 | 0.142 | 0.418 | 0.828 | 0.224 | 0.162 | 0.220 | 0.094 | 0.440 |
|  |  |  |  | 0.30 | 0.548 | 0.184 | 0.146 | 0.206 | 0.140 | 0.404 | 0.760 | 0.178 | 0.122 | 0.208 | 0.104 | 0.410 |
|  |  |  | 0.7 | 0.10 | 0.520 | 0.266 | 0.214 | 0.252 | 0.172 | 0.380 | 0.652 | 0.264 | 0.210 | 0.220 | 0.124 | 0.258 |
|  |  |  |  | 0.20 | 0.514 | 0.256 | 0.190 | 0.248 | 0.164 | 0.406 | 0.626 | 0.274 | 0.188 | 0.262 | 0.132 | 0.286 |
|  |  |  |  | 0.30 | 0.462 | 0.232 | 0.174 | 0.224 | 0.160 | 0.344 | 0.580 | 0.216 | 0.164 | 0.210 | 0.116 | 0.306 |
|  |  |  | 0.9 | 0.10 | 0.524 | 0.368 | 0.288 | 0.368 | 0.250 | 0.474 | 0.466 | 0.340 | 0.294 | 0.296 | 0.140 | 0.292 |
|  |  |  |  | 0.20 | 0.534 | 0.298 | 0.216 | 0.338 | 0.212 | 0.486 | 0.470 | 0.320 | 0.228 | 0.308 | 0.150 | 0.314 |
|  |  |  |  | 0.30 | 0.516 | 0.312 | 0.220 | 0.314 | 0.206 | 0.500 | 0.478 | 0.292 | 0.208 | 0.290 | 0.156 | 0.328 |
| 200 | 1 | 4 | 0.4 | 0.10 | 0.768 | 0.180 | 0.140 | 0.194 | 0.138 | 0.734 | 0.946 | 0.130 | 0.136 | 0.172 | 0.072 | 0.844 |
|  |  |  |  | 0.20 | 0.742 | 0.166 | 0.158 | 0.200 | 0.146 | 0.698 | 0.918 | 0.150 | 0.118 | 0.184 | 0.078 | 0.868 |
|  |  |  |  | 0.30 | 0.684 | 0.172 | 0.146 | 0.212 | 0.136 | 0.662 | 0.862 | 0.182 | 0.136 | 0.198 | 0.090 | 0.836 |
|  |  |  | 0.7 | 0.10 | 0.652 | 0.208 | 0.160 | 0.202 | 0.162 | 0.630 | 0.838 | 0.146 | 0.168 | 0.174 | 0.076 | 0.774 |
|  |  |  |  | 0.20 | 0.644 | 0.206 | 0.178 | 0.218 | 0.162 | 0.626 | 0.810 | 0.212 | 0.190 | 0.216 | 0.114 | 0.740 |
|  |  |  |  | 0.30 | 0.634 | 0.222 | 0.136 | 0.208 | 0.146 | 0.616 | 0.764 | 0.156 | 0.142 | 0.202 | 0.108 | 0.716 |
|  |  |  | 0.9 | 0.10 | 0.638 | 0.212 | 0.144 | 0.262 | 0.184 | 0.626 | 0.634 | 0.196 | 0.162 | 0.212 | 0.104 | 0.552 |
|  |  |  |  | 0.20 | 0.624 | 0.252 | 0.182 | 0.266 | 0.184 | 0.598 | 0.644 | 0.188 | 0.160 | 0.232 | 0.134 | 0.590 |
|  |  |  |  | 0.30 | 0.562 | 0.204 | 0.180 | 0.232 | 0.190 | 0.550 | 0.638 | 0.202 | 0.166 | 0.242 | 0.114 | 0.582 |
|  | 2 | 4 | 0.4 | 0.10 | 0.852 | 0.290 | 0.232 | 0.290 | 0.170 | 0.792 | 0.990 | 0.292 | 0.234 | 0.322 | 0.114 | 0.926 |
|  |  |  |  | 0.20 | 0.822 | 0.284 | 0.178 | 0.282 | 0.200 | 0.782 | 0.974 | 0.262 | 0.210 | 0.270 | 0.120 | 0.906 |
|  |  |  |  | 0.30 | 0.760 | 0.256 | 0.196 | 0.280 | 0.184 | 0.740 | 0.948 | 0.262 | 0.170 | 0.282 | 0.116 | 0.888 |
|  |  |  | 0.7 | 0.10 | 0.754 | 0.318 | 0.236 | 0.332 | 0.186 | 0.690 | 0.902 | 0.316 | 0.244 | 0.280 | 0.082 | 0.650 |
|  |  |  |  | 0.20 | 0.748 | 0.346 | 0.226 | 0.378 | 0.218 | 0.692 | 0.850 | 0.352 | 0.274 | 0.322 | 0.116 | 0.632 |
|  |  |  |  | 0.30 | 0.682 | 0.280 | 0.212 | 0.320 | 0.212 | 0.644 | 0.818 | 0.282 | 0.204 | 0.292 | 0.146 | 0.678 |
|  |  |  | 0.9 | 0.10 | 0.720 | 0.442 | 0.324 | 0.442 | 0.280 | 0.714 | 0.720 | 0.376 | 0.288 | 0.380 | 0.164 | 0.546 |
|  |  |  |  | 0.20 | 0.694 | 0.368 | 0.264 | 0.402 | 0.248 | 0.676 | 0.684 | 0.394 | 0.290 | 0.398 | 0.160 | 0.574 |
|  |  |  |  | 0.30 | 0.718 | 0.366 | 0.228 | 0.412 | 0.268 | 0.716 | 0.702 | 0.356 | 0.262 | 0.406 | 0.194 | 0.614 |
|  |  | 7 | 0.4 | 0.10 | 0.898 | 0.346 | 0.270 | 0.370 | 0.242 | 0.814 | 0.998 | 0.322 | 0.254 | 0.362 | 0.126 | 0.868 |
|  |  |  |  | 0.20 | 0.896 | 0.326 | 0.226 | 0.352 | 0.196 | 0.776 | 0.986 | 0.330 | 0.238 | 0.338 | 0.128 | 0.878 |
|  |  |  |  | 0.30 | 0.852 | 0.336 | 0.220 | 0.316 | 0.206 | 0.770 | 0.972 | 0.356 | 0.186 | 0.350 | 0.166 | 0.860 |
|  |  |  | 0.7 | 0.10 | 0.816 | 0.452 | 0.348 | 0.456 | 0.294 | 0.72 | 0.92 | 0.472 | 0.338 | 0.412 | 0.124 | 0.478 |
|  |  |  |  | 0.20 | 0.784 | 0.448 | 0.328 | 0.416 | 0.278 | 0.666 | 0.924 | 0.424 | 0.308 | 0.408 | 0.154 | 0.554 |
|  |  |  |  | 0.30 | 0.786 | 0.394 | 0.254 | 0.404 | 0.260 | 0.71 | 0.862 | 0.438 | 0.29 | 0.374 | 0.170 | 0.616 |
|  |  |  | 0.9 | 0.10 | 0.794 | 0.572 | 0.438 | 0.618 | 0.442 | 0.782 | 0.726 | 0.612 | 0.414 | 0.520 | 0.208 | 0.478 |
|  |  |  |  | 0.20 | 0.788 | 0.560 | 0.418 | 0.616 | 0.402 | 0.782 | 0.766 | 0.594 | 0.416 | 0.528 | 0.23 | 0.566 |
|  |  |  |  | 0.30 | 0.790 | 0.522 | 0.394 | 0.560 | 0.418 | 0.766 | 0.744 | 0.534 | 0.358 | 0.524 | 0.272 | 0.600 |
|  | 4 | 4 | 0.4 | 0.10 | 0.920 | 0.360 | 0.246 | 0.380 | 0.214 | 0.706 | 0.996 | 0.388 | 0.316 | 0.362 | 0.142 | 0.682 |
|  |  |  |  | 0.20 | 0.880 | 0.382 | 0.262 | 0.352 | 0.212 | 0.684 | 0.984 | 0.378 | 0.228 | 0.330 | 0.122 | 0.702 |
|  |  |  |  | 0.30 | 0.844 | 0.336 | 0.204 | 0.340 | 0.230 | 0.694 | 0.972 | 0.350 | 0.194 | 0.368 | 0.164 | 0.758 |
|  |  |  | 0.7 | 0.10 | 0.812 | 0.470 | 0.334 | 0.412 | 0.248 | 0.652 | 0.910 | 0.462 | 0.366 | 0.392 | 0.156 | 0.434 |
|  |  |  |  | 0.20 | 0.816 | 0.468 | 0.316 | 0.402 | 0.216 | 0.686 | 0.890 | 0.442 | 0.314 | 0.386 | 0.174 | 0.482 |
|  |  |  |  | 0.30 | 0.762 | 0.396 | 0.284 | 0.390 | 0.266 | 0.652 | 0.862 | 0.422 | 0.266 | 0.412 | 0.164 | 0.522 |
|  |  |  | 0.9 | 0.10 | 0.818 | 0.620 | 0.434 | 0.596 | 0.400 | 0.776 | 0.750 | 0.608 | 0.438 | 0.530 | 0.232 | 0.442 |
|  |  |  |  | 0.20 | 0.826 | 0.548 | 0.412 | 0.632 | 0.442 | 0.812 | 0.738 | 0.566 | 0.364 | 0.520 | 0.230 | 0.532 |
|  |  |  |  | 0.30 | 0.808 | 0.520 | 0.360 | 0.536 | 0.364 | 0.800 | 0.744 | 0.570 | 0.368 | 0.508 | 0.246 | 0.620 |
| 300 | 1 | 4 | 0.4 | 0.10 | 0.908 | 0.234 | 0.204 | 0.300 | 0.212 | 0.880 | 0.996 | 0.218 | 0.228 | 0.252 | 0.088 | 0.934 |
|  |  |  |  | 0.20 | 0.862 | 0.238 | 0.178 | 0.282 | 0.186 | 0.842 | 0.980 | 0.220 | 0.196 | 0.250 | 0.084 | 0.964 |
|  |  |  |  | 0.30 | 0.852 | 0.240 | 0.194 | 0.260 | 0.192 | 0.820 | 0.962 | 0.216 | 0.178 | 0.236 | 0.090 | 0.946 |
|  |  |  | 0.7 | 0.10 | 0.842 | 0.320 | 0.254 | 0.336 | 0.212 | 0.822 | 0.956 | 0.202 | 0.226 | 0.252 | 0.114 | 0.910 |
|  |  |  |  | 0.20 | 0.820 | 0.286 | 0.224 | 0.294 | 0.196 | 0.800 | 0.926 | 0.236 | 0.208 | 0.284 | 0.138 | 0.888 |
|  |  |  |  | 0.30 | 0.756 | 0.266 | 0.214 | 0.278 | 0.198 | 0.736 | 0.910 | 0.276 | 0.192 | 0.290 | 0.144 | 0.886 |
|  |  |  | 0.9 | 0.10 | 0.784 | 0.310 | 0.222 | 0.320 | 0.238 | 0.778 | 0.836 | 0.250 | 0.210 | 0.292 | 0.126 | 0.776 |
|  |  |  |  | 0.20 | 0.762 | 0.276 | 0.208 | 0.314 | 0.208 | 0.758 | 0.834 | 0.266 | 0.184 | 0.318 | 0.140 | 0.798 |
|  |  |  |  | 0.30 | 0.746 | 0.278 | 0.186 | 0.296 | 0.192 | 0.740 | 0.790 | 0.302 | 0.212 | 0.306 | 0.182 | 0.740 |
|  | 2 | 4 | 0.4 | 0.10 | 0.966 | 0.350 | 0.278 | 0.398 | 0.238 | 0.930 | 0.996 | 0.398 | 0.298 | 0.428 | 0.100 | 0.980 |
|  |  |  |  | 0.20 | 0.950 | 0.332 | 0.224 | 0.400 | 0.230 | 0.914 | 0.998 | 0.374 | 0.284 | 0.436 | 0.130 | 0.986 |
|  |  |  |  | 0.30 | 0.938 | 0.354 | 0.272 | 0.372 | 0.248 | 0.896 | 0.994 | 0.370 | 0.214 | 0.398 | 0.128 | 0.980 |
|  |  |  | 0.7 | 0.10 | 0.914 | 0.458 | 0.322 | 0.454 | 0.284 | 0.858 | 0.980 | 0.432 | 0.328 | 0.436 | 0.114 | 0.802 |
|  |  |  |  | 0.20 | 0.890 | 0.400 | 0.278 | 0.434 | 0.260 | 0.858 | 0.964 | 0.416 | 0.274 | 0.418 | 0.166 | 0.836 |
|  |  |  |  | 0.30 | 0.862 | 0.416 | 0.302 | 0.442 | 0.262 | 0.818 | 0.944 | 0.450 | 0.268 | 0.432 | 0.196 | 0.830 |
|  |  |  | 0.9 | 0.10 | 0.892 | 0.564 | 0.386 | 0.582 | 0.380 | 0.880 | 0.880 | 0.578 | 0.390 | 0.528 | 0.194 | 0.716 |
|  |  |  |  | 0.20 | 0.876 | 0.514 | 0.342 | 0.554 | 0.338 | 0.870 | 0.894 | 0.546 | 0.392 | 0.564 | 0.246 | 0.786 |
|  |  |  |  | 0.30 | 0.866 | 0.500 | 0.350 | 0.534 | 0.348 | 0.848 | 0.872 | 0.534 | 0.346 | 0.512 | 0.242 | 0.778 |
|  | 4 | 4 | 0.4 | 0.10 | 0.988 | 0.508 | 0.36 | 0.504 | 0.262 | 0.880 | 0.998 | 0.514 | 0.394 | 0.506 | 0.172 | 0.876 |
|  |  |  |  | 0.20 | 0.972 | 0.454 | 0.302 | 0.466 | 0.278 | 0.856 | 0.998 | 0.492 | 0.344 | 0.500 | 0.184 | 0.882 |
|  |  |  |  | 0.30 | 0.968 | 0.428 | 0.298 | 0.472 | 0.306 | 0.836 | 0.996 | 0.488 | 0.290 | 0.510 | 0.194 | 0.874 |
|  |  |  | 0.7 | 0.10 | 0.964 | 0.660 | 0.456 | 0.618 | 0.324 | 0.846 | 0.992 | 0.636 | 0.480 | 0.538 | 0.200 | 0.596 |
|  |  |  |  | 0.20 | 0.932 | 0.626 | 0.430 | 0.580 | 0.338 | 0.834 | 0.980 | 0.606 | 0.400 | 0.560 | 0.236 | 0.646 |
|  |  |  |  | 0.30 | 0.934 | 0.574 | 0.386 | 0.572 | 0.364 | 0.856 | 0.972 | 0.598 | 0.376 | 0.562 | 0.246 | 0.724 |
|  |  |  | 0.9 | 0.10 | 0.944 | 0.786 | 0.586 | 0.794 | 0.550 | 0.938 | 0.898 | 0.780 | 0.626 | 0.714 | 0.316 | 0.632 |
|  |  |  |  | 0.20 | 0.914 | 0.714 | 0.556 | 0.780 | 0.562 | 0.896 | 0.910 | 0.774 | 0.564 | 0.706 | 0.350 | 0.692 |
|  |  |  |  | 0.30 | 0.948 | 0.678 | 0.506 | 0.726 | 0.520 | 0.936 | 0.892 | 0.758 | 0.504 | 0.692 | 0.386 | 0.772 |

Methods compared are the Score and Mixed Model (SM), longitudinal Partial Credit Model (LPCM), Time to HRQoL score deterioration as compared to the baseline score (TTD baseline) or the best previous score (TTD best) and time until definitive deterioration of the HRQoL score as compared to the baseline score (TUDD baseline) or the best previous score (TUDD best) for different values of sample size (N), items (I), response category per item (J), correlations between HRQoL measure (ρ) and proportion of missing data (π).

**Table A3 -** Type I error rate of the test of interaction between treatment arm and time for datasets simulated with intermittent missing items and monotone missing data

|  |  |  |  |  | 5 measures | | | | | | 10 measures | | | | | |
| --- | --- | --- | --- | --- | --- | --- | --- | --- | --- | --- | --- | --- | --- | --- | --- | --- |
| N | I | J | ρ | π | SM | TTD baseline | TTD best | TUDD baseline | TUDD best | LPCM | SM | TTD baseline | TTD best | TUDD baseline | TUDD best | LPCM |
| 100 | 2 | 4 | 0.4 | 0.10 | 0.054 | 0.058 | 0.084 | 0.066 | 0.076 | 0.064 | 0.042 | 0.046 | 0.040 | 0.056 | 0.056 | 0.052 |
|  |  |  |  | 0.20 | 0.058 | 0.058 | 0.058 | 0.062 | 0.064 | 0.062 | 0.056 | 0.052 | 0.056 | 0.052 | 0.060 | 0.064 |
|  |  |  |  | 0.30 | 0.070 | 0.068 | 0.052 | 0.070 | 0.054 | 0.066 | 0.060 | 0.048 | 0.054 | 0.058 | 0.046 | 0.064 |
|  |  |  | 0.7 | 0.10 | 0.054 | 0.038 | 0.046 | 0.064 | 0.054 | 0.060 | 0.044 | 0.042 | 0.036 | 0.046 | 0.048 | 0.044 |
|  |  |  |  | 0.20 | 0.060 | 0.038 | 0.052 | 0.058 | 0.078 | 0.066 | 0.060 | 0.040 | 0.054 | 0.056 | 0.066 | 0.044 |
|  |  |  |  | 0.30 | 0.062 | 0.042 | 0.042 | 0.052 | 0.046 | 0.066 | 0.046 | 0.060 | 0.054 | 0.06 | 0.056 | 0.034 |
|  |  |  | 0.9 | 0.10 | 0.050 | 0.050 | 0.054 | 0.046 | 0.060 | 0.052 | 0.056 | 0.048 | 0.054 | 0.052 | 0.072 | 0.052 |
|  |  |  |  | 0.20 | 0.052 | 0.040 | 0.040 | 0.056 | 0.068 | 0.058 | 0.064 | 0.040 | 0.048 | 0.058 | 0.050 | 0.044 |
|  |  |  |  | 0.30 | 0.042 | 0.044 | 0.048 | 0.044 | 0.064 | 0.048 | 0.068 | 0.050 | 0.050 | 0.054 | 0.066 | 0.072 |
|  | 4 | 4 | 0.4 | 0.10 | 0.050 | 0.056 | 0.064 | 0.066 | 0.058 | 0.054 | 0.068 | 0.078 | 0.072 | 0.058 | 0.056 | 0.046 |
|  |  |  |  | 0.20 | 0.052 | 0.064 | 0.070 | 0.066 | 0.064 | 0.066 | 0.048 | 0.066 | 0.050 | 0.068 | 0.048 | 0.048 |
|  |  |  |  | 0.30 | 0.060 | 0.060 | 0.058 | 0.062 | 0.060 | 0.086 | 0.058 | 0.060 | 0.046 | 0.072 | 0.074 | 0.048 |
|  |  |  | 0.7 | 0.10 | 0.060 | 0.054 | 0.056 | 0.060 | 0.050 | 0.068 | 0.056 | 0.060 | 0.066 | 0.060 | 0.06 | 0.056 |
|  |  |  |  | 0.20 | 0.064 | 0.058 | 0.046 | 0.056 | 0.080 | 0.068 | 0.042 | 0.070 | 0.058 | 0.042 | 0.050 | 0.056 |
|  |  |  |  | 0.30 | 0.064 | 0.042 | 0.044 | 0.066 | 0.064 | 0.064 | 0.052 | 0.046 | 0.054 | 0.054 | 0.060 | 0.046 |
|  |  |  | 0.9 | 0.10 | 0.052 | 0.058 | 0.076 | 0.076 | 0.050 | 0.060 | 0.064 | 0.032 | 0.056 | 0.062 | 0.066 | 0.046 |
|  |  |  |  | 0.20 | 0.038 | 0.046 | 0.048 | 0.044 | 0.044 | 0.036 | 0.064 | 0.058 | 0.058 | 0.046 | 0.076 | 0.052 |
|  |  |  |  | 0.30 | 0.082 | 0.062 | 0.074 | 0.066 | 0.072 | 0.068 | 0.050 | 0.056 | 0.048 | 0.058 | 0.050 | 0.058 |
| 200 | 2 | 4 | 0.4 | 0.10 | 0.048 | 0.058 | 0.044 | 0.042 | 0.050 | 0.044 | 0.048 | 0.046 | 0.062 | 0.056 | 0.042 | 0.044 |
|  |  |  |  | 0.20 | 0.044 | 0.064 | 0.064 | 0.054 | 0.050 | 0.044 | 0.048 | 0.058 | 0.044 | 0.042 | 0.044 | 0.044 |
|  |  |  |  | 0.30 | 0.046 | 0.064 | 0.058 | 0.060 | 0.062 | 0.050 | 0.064 | 0.066 | 0.068 | 0.068 | 0.058 | 0.064 |
|  |  |  | 0.7 | 0.10 | 0.062 | 0.060 | 0.078 | 0.052 | 0.054 | 0.052 | 0.040 | 0.064 | 0.060 | 0.052 | 0.05 | 0.036 |
|  |  |  |  | 0.20 | 0.054 | 0.056 | 0.068 | 0.056 | 0.072 | 0.056 | 0.044 | 0.042 | 0.034 | 0.046 | 0.066 | 0.046 |
|  |  |  |  | 0.30 | 0.088 | 0.056 | 0.062 | 0.056 | 0.052 | 0.080 | 0.044 | 0.044 | 0.056 | 0.058 | 0.066 | 0.058 |
|  |  |  | 0.9 | 0.10 | 0.052 | 0.052 | 0.056 | 0.056 | 0.046 | 0.056 | 0.046 | 0.048 | 0.064 | 0.046 | 0.06 | 0.048 |
|  |  |  |  | 0.20 | 0.060 | 0.056 | 0.042 | 0.058 | 0.054 | 0.064 | 0.042 | 0.044 | 0.050 | 0.058 | 0.058 | 0.032 |
|  |  |  |  | 0.30 | 0.024 | 0.060 | 0.048 | 0.052 | 0.042 | 0.040 | 0.046 | 0.068 | 0.062 | 0.052 | 0.040 | 0.052 |
|  |  | 7 | 0.4 | 0.10 | 0.060 | 0.056 | 0.046 | 0.066 | 0.048 | 0.058 | 0.056 | 0.076 | 0.070 | 0.052 | 0.058 | 0.046 |
|  |  |  |  | 0.20 | 0.052 | 0.054 | 0.032 | 0.062 | 0.056 | 0.044 | 0.046 | 0.052 | 0.052 | 0.056 | 0.064 | 0.048 |
|  |  |  |  | 0.30 | 0.050 | 0.032 | 0.046 | 0.056 | 0.056 | 0.054 | 0.042 | 0.052 | 0.056 | 0.052 | 0.068 | 0.032 |
|  |  |  | 0.7 | 0.10 | 0.036 | 0.064 | 0.048 | 0.064 | 0.044 | 0.038 | 0.042 | 0.050 | 0.058 | 0.062 | 0.056 | 0.048 |
|  |  |  |  | 0.20 | 0.060 | 0.060 | 0.072 | 0.046 | 0.046 | 0.058 | 0.036 | 0.066 | 0.052 | 0.058 | 0.066 | 0.032 |
|  |  |  |  | 0.30 | 0.048 | 0.050 | 0.062 | 0.050 | 0.054 | 0.052 | 0.046 | 0.038 | 0.052 | 0.062 | 0.050 | 0.042 |
|  |  |  | 0.9 | 0.10 | 0.056 | 0.048 | 0.046 | 0.046 | 0.046 | 0.050 | 0.046 | 0.052 | 0.052 | 0.048 | 0.054 | 0.044 |
|  |  |  |  | 0.20 | 0.050 | 0.054 | 0.058 | 0.052 | 0.050 | 0.044 | 0.046 | 0.042 | 0.056 | 0.046 | 0.066 | 0.058 |
|  |  |  |  | 0.30 | 0.05 | 0.058 | 0.048 | 0.042 | 0.052 | 0.058 | 0.052 | 0.038 | 0.054 | 0.068 | 0.050 | 0.052 |
|  | 4 | 4 | 0.4 | 0.10 | 0.076 | 0.056 | 0.046 | 0.042 | 0.044 | 0.040 | 0.050 | 0.058 | 0.048 | 0.050 | 0.054 | 0.040 |
|  |  |  |  | 0.20 | 0.044 | 0.064 | 0.072 | 0.056 | 0.062 | 0.034 | 0.042 | 0.048 | 0.060 | 0.054 | 0.070 | 0.050 |
|  |  |  |  | 0.30 | 0.050 | 0.054 | 0.048 | 0.050 | 0.036 | 0.040 | 0.048 | 0.052 | 0.046 | 0.062 | 0.042 | 0.042 |
|  |  |  | 0.7 | 0.10 | 0.056 | 0.054 | 0.042 | 0.052 | 0.052 | 0.046 | 0.050 | 0.046 | 0.046 | 0.066 | 0.056 | 0.060 |
|  |  |  |  | 0.20 | 0.042 | 0.046 | 0.048 | 0.046 | 0.050 | 0.038 | 0.060 | 0.052 | 0.042 | 0.048 | 0.046 | 0.070 |
|  |  |  |  | 0.30 | 0.060 | 0.066 | 0.044 | 0.060 | 0.036 | 0.036 | 0.042 | 0.062 | 0.064 | 0.066 | 0.052 | 0.042 |
|  |  |  | 0.9 | 0.10 | 0.058 | 0.054 | 0.056 | 0.056 | 0.058 | 0.062 | 0.030 | 0.038 | 0.048 | 0.042 | 0.044 | 0.036 |
|  |  |  |  | 0.20 | 0.064 | 0.062 | 0.054 | 0.064 | 0.054 | 0.070 | 0.050 | 0.076 | 0.070 | 0.062 | 0.052 | 0.044 |
|  |  |  |  | 0.30 | 0.048 | 0.036 | 0.030 | 0.028 | 0.028 | 0.048 | 0.052 | 0.064 | 0.066 | 0.058 | 0.042 | 0.042 |
| 300 | 2 | 4 | 0.4 | 0.10 | 0.042 | 0.046 | 0.050 | 0.054 | 0.052 | 0.048 | 0.040 | 0.056 | 0.042 | 0.062 | 0.052 | 0.046 |
|  |  |  |  | 0.20 | 0.042 | 0.052 | 0.052 | 0.044 | 0.030 | 0.046 | 0.034 | 0.036 | 0.040 | 0.046 | 0.042 | 0.052 |
|  |  |  |  | 0.30 | 0.036 | 0.048 | 0.056 | 0.048 | 0.048 | 0.050 | 0.050 | 0.032 | 0.040 | 0.046 | 0.046 | 0.050 |
|  |  |  | 0.7 | 0.10 | 0.054 | 0.058 | 0.062 | 0.056 | 0.052 | 0.058 | 0.034 | 0.060 | 0.054 | 0.040 | 0.044 | 0.038 |
|  |  |  |  | 0.20 | 0.038 | 0.044 | 0.044 | 0.058 | 0.060 | 0.042 | 0.046 | 0.060 | 0.050 | 0.058 | 0.064 | 0.046 |
|  |  |  |  | 0.30 | 0.050 | 0.034 | 0.040 | 0.038 | 0.048 | 0.056 | 0.036 | 0.050 | 0.048 | 0.028 | 0.056 | 0.038 |
|  |  |  | 0.9 | 0.10 | 0.044 | 0.044 | 0.060 | 0.052 | 0.050 | 0.042 | 0.048 | 0.042 | 0.048 | 0.048 | 0.054 | 0.056 |
|  |  |  |  | 0.20 | 0.036 | 0.036 | 0.048 | 0.042 | 0.044 | 0.044 | 0.046 | 0.050 | 0.038 | 0.034 | 0.034 | 0.038 |
|  |  |  |  | 0.30 | 0.040 | 0.048 | 0.050 | 0.036 | 0.040 | 0.046 | 0.046 | 0.048 | 0.046 | 0.054 | 0.052 | 0.058 |
|  | 4 | 4 | 0.4 | 0.10 | 0.038 | 0.038 | 0.044 | 0.042 | 0.044 | 0.048 | 0.036 | 0.064 | 0.070 | 0.046 | 0.054 | 0.046 |
|  |  |  |  | 0.20 | 0.048 | 0.056 | 0.060 | 0.054 | 0.062 | 0.058 | 0.028 | 0.064 | 0.066 | 0.052 | 0.050 | 0.038 |
|  |  |  |  | 0.30 | 0.044 | 0.054 | 0.054 | 0.058 | 0.052 | 0.066 | 0.052 | 0.042 | 0.038 | 0.054 | 0.056 | 0.044 |
|  |  |  | 0.7 | 0.10 | 0.040 | 0.054 | 0.058 | 0.050 | 0.044 | 0.046 | 0.054 | 0.038 | 0.050 | 0.048 | 0.060 | 0.042 |
|  |  |  |  | 0.20 | 0.038 | 0.064 | 0.058 | 0.048 | 0.070 | 0.044 | 0.042 | 0.042 | 0.050 | 0.042 | 0.046 | 0.060 |
|  |  |  |  | 0.30 | 0.038 | 0.052 | 0.048 | 0.040 | 0.056 | 0.062 | 0.042 | 0.04 | 0.048 | 0.056 | 0.048 | 0.042 |
|  |  |  | 0.9 | 0.10 | 0.042 | 0.046 | 0.052 | 0.040 | 0.054 | 0.044 | 0.036 | 0.036 | 0.056 | 0.05 | 0.050 | 0.040 |
|  |  |  |  | 0.20 | 0.034 | 0.038 | 0.060 | 0.050 | 0.050 | 0.022 | 0.040 | 0.038 | 0.060 | 0.050 | 0.056 | 0.036 |
|  |  |  |  | 0.30 | 0.050 | 0.062 | 0.064 | 0.042 | 0.046 | 0.056 | 0.054 | 0.042 | 0.064 | 0.048 | 0.056 | 0.058 |

Methods compared are the Score and Mixed Model (SM), longitudinal Partial Credit Model (LPCM), Time to HRQoL score deterioration as compared to the baseline score (TTD baseline) or the best previous score (TTD best) and time until definitive deterioration of the HRQoL score as compared to the baseline score (TUDD baseline) or the best previous score (TUDD best) for different values of sample size (N), items (I), response category per item (J), correlations between HRQoL measure (ρ) and proportion of missing data (π).

**Table A4 -** Power of the test of interaction between treatment arm and time for the datasets simulated with intermittent missing items and monotone missing data

|  |  |  |  |  | 5 measures | | | | | | 10 measures | | | | | |
| --- | --- | --- | --- | --- | --- | --- | --- | --- | --- | --- | --- | --- | --- | --- | --- | --- |
| N | I | J | ρ | π | SM | TTD baseline | TTD best | TUDD baseline | TUDD best | LPCM | SM | TTD baseline | TTD best | TUDD baseline | TUDD best | LPCM |
| 100 | 2 | 4 | 0.4 | 0.10 | 0.582 | 0.180 | 0.142 | 0.178 | 0.116 | 0.524 | 0.840 | 0.176 | 0.158 | 0.170 | 0.104 | 0.672 |
|  |  |  |  | 0.20 | 0.534 | 0.186 | 0.146 | 0.218 | 0.146 | 0.476 | 0.762 | 0.174 | 0.150 | 0.182 | 0.096 | 0.638 |
|  |  |  |  | 0.30 | 0.516 | 0.160 | 0.134 | 0.168 | 0.112 | 0.478 | 0.724 | 0.154 | 0.136 | 0.136 | 0.058 | 0.590 |
|  |  |  | 0.7 | 0.10 | 0.476 | 0.194 | 0.168 | 0.198 | 0.156 | 0.404 | 0.600 | 0.184 | 0.158 | 0.158 | 0.082 | 0.354 |
|  |  |  |  | 0.20 | 0.446 | 0.182 | 0.128 | 0.188 | 0.124 | 0.394 | 0.578 | 0.200 | 0.178 | 0.178 | 0.064 | 0.368 |
|  |  |  |  | 0.30 | 0.418 | 0.192 | 0.144 | 0.176 | 0.132 | 0.370 | 0.552 | 0.156 | 0.116 | 0.196 | 0.088 | 0.422 |
|  |  |  | 0.9 | 0.10 | 0.428 | 0.246 | 0.192 | 0.270 | 0.172 | 0.420 | 0.426 | 0.252 | 0.212 | 0.218 | 0.112 | 0.324 |
|  |  |  |  | 0.20 | 0.442 | 0.256 | 0.178 | 0.252 | 0.176 | 0.420 | 0.432 | 0.230 | 0.182 | 0.230 | 0.086 | 0.344 |
|  |  |  |  | 0.30 | 0.434 | 0.256 | 0.200 | 0.256 | 0.194 | 0.412 | 0.412 | 0.218 | 0.180 | 0.232 | 0.124 | 0.346 |
|  | 4 | 4 | 0.4 | 0.10 | 0.592 | 0.134 | 0.108 | 0.178 | 0.148 | 0.396 | 0.858 | 0.19 | 0.154 | 0.202 | 0.102 | 0.412 |
|  |  |  |  | 0.20 | 0.536 | 0.162 | 0.134 | 0.198 | 0.148 | 0.446 | 0.780 | 0.188 | 0.116 | 0.190 | 0.108 | 0.476 |
|  |  |  |  | 0.30 | 0.454 | 0.118 | 0.094 | 0.164 | 0.130 | 0.410 | 0.628 | 0.146 | 0.102 | 0.160 | 0.106 | 0.468 |
|  |  |  | 0.7 | 0.10 | 0.472 | 0.228 | 0.156 | 0.238 | 0.160 | 0.356 | 0.618 | 0.212 | 0.166 | 0.230 | 0.140 | 0.234 |
|  |  |  |  | 0.20 | 0.486 | 0.212 | 0.164 | 0.204 | 0.164 | 0.398 | 0.608 | 0.188 | 0.130 | 0.186 | 0.130 | 0.268 |
|  |  |  |  | 0.30 | 0.404 | 0.164 | 0.146 | 0.162 | 0.154 | 0.408 | 0.502 | 0.162 | 0.110 | 0.218 | 0.114 | 0.342 |
|  |  |  | 0.9 | 0.10 | 0.532 | 0.278 | 0.202 | 0.334 | 0.224 | 0.472 | 0.472 | 0.344 | 0.234 | 0.312 | 0.150 | 0.248 |
|  |  |  |  | 0.20 | 0.502 | 0.210 | 0.166 | 0.244 | 0.172 | 0.470 | 0.474 | 0.240 | 0.144 | 0.272 | 0.144 | 0.302 |
|  |  |  |  | 0.30 | 0.460 | 0.200 | 0.144 | 0.238 | 0.166 | 0.508 | 0.478 | 0.232 | 0.180 | 0.238 | 0.148 | 0.342 |
| 200 | 2 | 4 | 0.4 | 0.10 | 0.864 | 0.290 | 0.226 | 0.278 | 0.180 | 0.818 | 0.988 | 0.270 | 0.210 | 0.306 | 0.092 | 0.938 |
|  |  |  |  | 0.20 | 0.826 | 0.274 | 0.232 | 0.288 | 0.178 | 0.744 | 0.982 | 0.288 | 0.214 | 0.272 | 0.082 | 0.924 |
|  |  |  |  | 0.30 | 0.802 | 0.270 | 0.206 | 0.292 | 0.178 | 0.772 | 0.960 | 0.272 | 0.214 | 0.292 | 0.092 | 0.900 |
|  |  |  | 0.7 | 0.10 | 0.786 | 0.328 | 0.256 | 0.354 | 0.212 | 0.728 | 0.892 | 0.320 | 0.270 | 0.312 | 0.076 | 0.618 |
|  |  |  |  | 0.20 | 0.726 | 0.320 | 0.264 | 0.354 | 0.198 | 0.684 | 0.840 | 0.336 | 0.264 | 0.340 | 0.106 | 0.644 |
|  |  |  |  | 0.30 | 0.696 | 0.312 | 0.214 | 0.318 | 0.162 | 0.650 | 0.824 | 0.372 | 0.282 | 0.286 | 0.138 | 0.670 |
|  |  |  | 0.9 | 0.10 | 0.722 | 0.722 | 0.340 | 0.454 | 0.290 | 0.724 | 0.692 | 0.378 | 0.284 | 0.374 | 0.136 | 0.528 |
|  |  |  |  | 0.20 | 0.686 | 0.386 | 0.268 | 0.440 | 0.270 | 0.716 | 0.672 | 0.394 | 0.276 | 0.410 | 0.128 | 0.584 |
|  |  |  |  | 0.30 | 0.670 | 0.404 | 0.294 | 0.436 | 0.270 | 0.692 | 0.686 | 0.350 | 0.252 | 0.362 | 0.162 | 0.618 |
|  |  | 7 | 0.4 | 0.10 | 0.922 | 0.386 | 0.278 | 0.386 | 0.228 | 0.808 | 0.994 | 0.408 | 0.324 | 0.382 | 0.112 | 0.880 |
|  |  |  |  | 0.20 | 0.906 | 0.376 | 0.274 | 0.386 | 0.262 | 0.812 | 0.990 | 0.370 | 0.304 | 0.382 | 0.136 | 0.900 |
|  |  |  |  | 0.30 | 0.854 | 0.350 | 0.288 | 0.340 | 0.232 | 0.778 | 0.970 | 0.364 | 0.292 | 0.382 | 0.108 | 0.888 |
|  |  |  | 0.7 | 0.10 | 0.816 | 0.460 | 0.370 | 0.444 | 0.302 | 0.702 | 0.912 | 0.498 | 0.366 | 0.38 | 0.124 | 0.516 |
|  |  |  |  | 0.20 | 0.792 | 0.454 | 0.372 | 0.448 | 0.268 | 0.714 | 0.904 | 0.464 | 0.384 | 0.404 | 0.156 | 0.606 |
|  |  |  |  | 0.30 | 0.794 | 0.470 | 0.350 | 0.452 | 0.270 | 0.724 | 0.88 | 0.442 | 0.318 | 0.396 | 0.148 | 0.628 |
|  |  |  | 0.9 | 0.10 | 0.784 | 0.594 | 0.484 | 0.594 | 0.440 | 0.78 | 0.740 | 0.614 | 0.510 | 0.526 | 0.188 | 0.510 |
|  |  |  |  | 0.20 | 0.794 | 0.596 | 0.418 | 0.632 | 0.434 | 0.818 | 0.724 | 0.58 | 0.468 | 0.514 | 0.216 | 0.546 |
|  |  |  |  | 0.30 | 0.800 | 0.558 | 0.404 | 0.616 | 0.428 | 0.800 | 0.734 | 0.594 | 0.440 | 0.556 | 0.24 | 0.618 |
|  | 4 | 4 | 0.4 | 0.10 | 0.888 | 0.320 | 0.200 | 0.366 | 0.218 | 0.716 | 0.990 | 0.346 | 0.206 | 0.362 | 0.124 | 0.704 |
|  |  |  |  | 0.20 | 0.840 | 0.240 | 0.148 | 0.264 | 0.180 | 0.698 | 0.972 | 0.294 | 0.148 | 0.324 | 0.158 | 0.748 |
|  |  |  |  | 0.30 | 0.784 | 0.222 | 0.174 | 0.254 | 0.188 | 0.698 | 0.912 | 0.252 | 0.136 | 0.28 | 0.152 | 0.770 |
|  |  |  | 0.7 | 0.10 | 0.818 | 0.414 | 0.278 | 0.410 | 0.258 | 0.674 | 0.912 | 0.396 | 0.248 | 0.364 | 0.154 | 0.426 |
|  |  |  |  | 0.20 | 0.738 | 0.310 | 0.220 | 0.334 | 0.224 | 0.652 | 0.868 | 0.322 | 0.214 | 0.350 | 0.176 | 0.484 |
|  |  |  |  | 0.30 | 0.716 | 0.282 | 0.202 | 0.280 | 0.210 | 0.712 | 0.800 | 0.266 | 0.142 | 0.316 | 0.180 | 0.540 |
|  |  |  | 0.9 | 0.10 | 0.844 | 0.538 | 0.388 | 0.568 | 0.388 | 0.806 | 0.768 | 0.580 | 0.360 | 0.506 | 0.234 | 0.460 |
|  |  |  |  | 0.20 | 0.778 | 0.442 | 0.290 | 0.488 | 0.340 | 0.776 | 0.790 | 0.436 | 0.266 | 0.488 | 0.230 | 0.548 |
|  |  |  |  | 0.30 | 0.728 | 0.328 | 0.248 | 0.378 | 0.278 | 0.786 | 0.708 | 0.412 | 0.230 | 0.414 | 0.226 | 0.584 |
| 300 | 2 | 4 | 0.4 | 0.10 | 0.970 | 0.430 | 0.324 | 0.432 | 0.240 | 0.936 | 0.998 | 0.386 | 0.340 | 0.374 | 0.09 | 0.984 |
|  |  |  |  | 0.20 | 0.956 | 0.374 | 0.276 | 0.394 | 0.250 | 0.924 | 0.996 | 0.456 | 0.332 | 0.444 | 0.096 | 0.982 |
|  |  |  |  | 0.30 | 0.934 | 0.426 | 0.296 | 0.428 | 0.254 | 0.884 | 0.994 | 0.404 | 0.302 | 0.400 | 0.088 | 0.984 |
|  |  |  | 0.7 | 0.10 | 0.922 | 0.498 | 0.332 | 0.510 | 0.304 | 0.882 | 0.978 | 0.466 | 0.368 | 0.466 | 0.130 | 0.806 |
|  |  |  |  | 0.20 | 0.894 | 0.484 | 0.356 | 0.510 | 0.322 | 0.870 | 0.970 | 0.452 | 0.322 | 0.496 | 0.156 | 0.866 |
|  |  |  |  | 0.30 | 0.866 | 0.458 | 0.376 | 0.466 | 0.310 | 0.818 | 0.956 | 0.440 | 0.314 | 0.424 | 0.158 | 0.866 |
|  |  |  | 0.9 | 0.10 | 0.880 | 0.588 | 0.438 | 0.610 | 0.376 | 0.866 | 0.878 | 0.538 | 0.424 | 0.508 | 0.184 | 0.702 |
|  |  |  |  | 0.20 | 0.860 | 0.568 | 0.372 | 0.596 | 0.372 | 0.860 | 0.852 | 0.532 | 0.410 | 0.480 | 0.176 | 0.740 |
|  |  |  |  | 0.30 | 0.854 | 0.520 | 0.382 | 0.592 | 0.386 | 0.850 | 0.870 | 0.498 | 0.360 | 0.520 | 0.202 | 0.802 |
|  | 4 | 4 | 0.4 | 0.10 | 0.978 | 0.438 | 0.278 | 0.474 | 0.266 | 0.894 | 0.998 | 0.476 | 0.26 | 0.49 | 0.186 | 0.856 |
|  |  |  |  | 0.20 | 0.950 | 0.372 | 0.234 | 0.394 | 0.250 | 0.876 | 0.992 | 0.376 | 0.190 | 0.446 | 0.212 | 0.876 |
|  |  |  |  | 0.30 | 0.902 | 0.318 | 0.244 | 0.346 | 0.250 | 0.878 | 0.978 | 0.336 | 0.192 | 0.422 | 0.234 | 0.900 |
|  |  |  | 0.7 | 0.10 | 0.934 | 0.560 | 0.394 | 0.562 | 0.338 | 0.828 | 0.990 | 0.584 | 0.364 | 0.536 | 0.226 | 0.604 |
|  |  |  |  | 0.20 | 0.918 | 0.476 | 0.320 | 0.514 | 0.320 | 0.850 | 0.966 | 0.476 | 0.278 | 0.482 | 0.266 | 0.646 |
|  |  |  |  | 0.30 | 0.836 | 0.354 | 0.284 | 0.370 | 0.268 | 0.840 | 0.928 | 0.430 | 0.252 | 0.45 | 0.238 | 0.732 |
|  |  |  | 0.9 | 0.10 | 0.944 | 0.686 | 0.490 | 0.736 | 0.526 | 0.918 | 0.916 | 0.720 | 0.466 | 0.704 | 0.340 | 0.628 |
|  |  |  |  | 0.20 | 0.926 | 0.564 | 0.412 | 0.592 | 0.414 | 0.922 | 0.916 | 0.636 | 0.364 | 0.678 | 0.372 | 0.758 |
|  |  |  |  | 0.30 | 0.880 | 0.474 | 0.344 | 0.538 | 0.398 | 0.908 | 0.904 | 0.516 | 0.308 | 0.544 | 0.352 | 0.782 |

Methods compared are the Score and Mixed Model (SM), longitudinal Partial Credit Model (LPCM), Time to HRQoL score deterioration as compared to the baseline score (TTD baseline) or the best previous score (TTD best) and time until definitive deterioration of the HRQoL score as compared to the baseline score (TUDD baseline) or the best previous score (TUDD best) for different values of sample size (N), items (I), response category per item (J), correlations between HRQoL measure (ρ) and proportion of missing data (π)
